# Silk fibroin hydrogels for biomedical applications

**DOI:** 10.1002/SMMD.20220011

**Published:** 2022-12-23

**Authors:** Hui Zhang, Dongyu Xu, Yong Zhang, Minli Li, Renjie Chai

**Affiliations:** ^1^ State Key Laboratory of Bioelectronics Department of Otolaryngology Head and Neck Surgery Zhongda Hospital School of Life Science and Technology Jiangsu Province High‐Tech Key Laboratory for Bio‐Medical Research Southeast University Nanjing China; ^2^ School of Biological Science and Medical Engineering Southeast University Nanjing China; ^3^ School of Physics Southeast University Nanjing China; ^4^ Co‐innovation Center of Neuroregeneration Nantong University Nantong China; ^5^ Department of Otorhinolaryngology‐Head and Neck Surgery Affiliated Drum Tower Hospital of Nanjing University Medical School Nanjing China; ^6^ Department of Otolaryngology Head and Neck Surgery Sichuan Provincial People's Hospital University of Electronic Science and Technology of China Chengdu China; ^7^ Institute for Stem Cell and Regeneration Chinese Academy of Sciences Beijing China; ^8^ Beijing Key Laboratory of Neural Regeneration and Repair Capital Medical University Beijing China

**Keywords:** drug delivery, hydrogel, silk fibroin, tissue engineering, wearable sensors

## Abstract

Silk fibroin hydrogels occupy an essential position in the biomedical field due to their remarkable biological properties, excellent mechanical properties, flexible processing properties, as well as abundant sources and low cost. Herein, we introduce the unique structures and physicochemical characteristics of silk fibroin, including mechanical properties, biocompatibility, and biodegradability. Then, various preparation strategies of silk fibroin hydrogels are summarized, which can be divided into physical cross‐linking and chemical cross‐linking. Emphatically, the applications of silk fibroin hydrogel biomaterials in various biomedical fields, including tissue engineering, drug delivery, and wearable sensors, are systematically summarized. At last, the challenges and future prospects of silk fibroin hydrogels in biomedical applications are discussed.

## Graphical abstract

Benefiting from the remarkable biological and physicochemical properties, silk fibroin hydrogels occupy a vital position in the biomedical field. We review the recent developments of silk fibroin‐derived hydrogels, outline their unique structures and important properties, and highlight their applications in various biomedical fields, including tissue engineering, drug delivery, and wearable sensors.

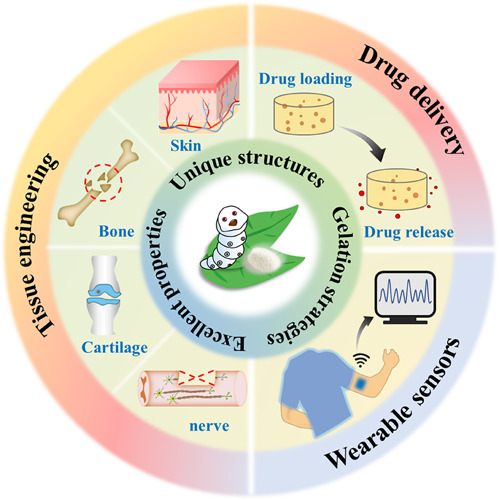

1

**Key points**

- The remarkable physiochemical and biological properties of silk fibroin hydrogels are introduced.
- The various preparation strategies of silk fibroin‐derived hydrogels are comprehensively summarized.
- The applications of silk fibroin‐derived hydrogels in multifarious biomedical fields are emphasized.

## 2 INTRODUCTION

Hydrogels are typical water molecule‐rich polymers with three‐dimensional (3D) and cross‐linked network structures.^1^, ^2^, ^3^ Such a network system possesses the ability to absorb a large number of water molecules and swell while maintaining a certain shape in a liquid environment for a long time.^4^, ^5^ With a porous structure resembling the extracellular matrix (ECM), hydrogels allow the circulation and exchange of nutrients and wastes, thus indicating positive implications for cell adhesion, proliferation, migration, as well as delivery of small or macro‐biomolecules.^6^, ^7^, ^8^ Of note, through the design of the polymer chain structure and the introduction of additives, various excellent properties such as high mechanical strength, suitable degradation rate, and stimuli responsiveness can be imparted to the hydrogels. Therefore, hydrogels show unparalleled application potential in multifarious fields, such as cell culture, tissue engineering, drug delivery, and other fields. Compared with synthetic polymer hydrogels, natural polymer hydrogels (such as collagen, alginate, chitosan, and cellulose) possess unique natural properties similar to tissue structures and satisfactory biocompatibility, thus receiving extensive attention in biomedicine, especially tissue engineering.^9^, ^10^, ^11^, ^12^ Although with promising applied results, these polymer hydrogels still show some drawbacks such as poor mechanical performances and high cost.

Silk with abundant output and remarkable mechanical properties has been utilized for thousands of years, and *Bombyx mori* silkworms are deemed as the world's leading producer.^13^, ^14^, ^15^, ^16^, ^17^, ^18^ Raw silk comprises two parallel silk fibroin fibers with sericin glued to their surface. By degumming raw silk (removing sericin), the obtained silk fibroin as the natural green protein material exhibits excellent biocompatibility, biodegradability, non‐toxicity, thermal stability, together with attractive mechanical strength and toughness, which outperforms many natural and synthetic fibers, playing a pivotal role in the biomedical field.^14^ Silk fibroin hydrogels can be manufactured by treating the dissolved degummed fibers with various physical or chemical means. The resultant silk fibroin hydrogels demonstrate positive interactions with biological systems and advantageous performances for cell activities. Particularly, the controllable molecular structures impart tunable mechanical properties and degradation rates to silk fibroin hydrogels, which enables them to adapt to various biological applications flexibly.^13^, ^19^ Furthermore, there is growing interest in introducing more functions to silk fibroin hydrogels while retaining their inherent properties. Silk fibroin hydrogels, together with their derived composite hydrogels, are endowed with improved mechanical strength, high elasticity, injectability, and environmental sensitivity, resulting in a series of satisfactory research results.

Herein, we summarize the recent research progress of silk fibroin hydrogels in the biomedical field. First, silk fibroin's structures and physicochemical properties are outlined. Then, classified according to the cross‐linking mechanisms, two different gelation methods of silk fibroin hydrogels are introduced. We highlight the current application status of silk fibroin hydrogels for tissue engineering, drug delivery, and as biosensors. Finally, we propose the challenges and development prospects of silk fibroin hydrogels in the biomedical field.

## 3 STRUCTURES AND PROPERTIES OF SILK FIBROIN

### 3.1 Structures

**FIGURE 1:**
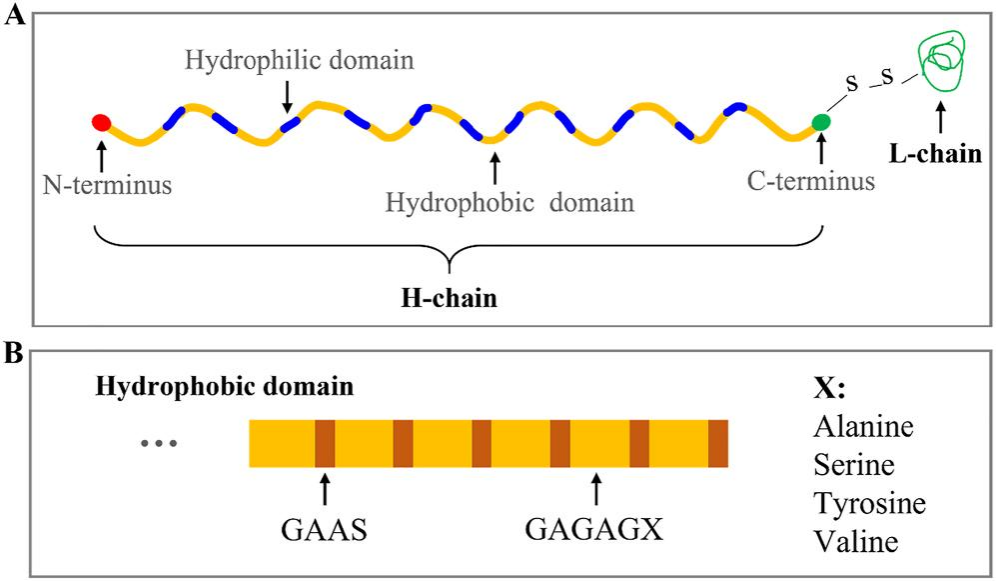
(A) Schematic of silk fibroin chemical structure. (B) Schematic illustration of repeating domain composition.

Silk fibroin contains a heavy (H‐) chain peptide (390 kDa) and a light (L‐) chain peptide (26 kDa) in a ratio of 1:1, wherein the L‐chain is linked to the C‐terminus of the H‐chain through a single disulfide.^20^ Further, the H‐L complex connects with the glycoprotein (P25, 25 kDa) by six to one through the hydrophobic interactions to form a micellar unit.^21^ Notably, the formation of *ß*‐sheet crystallites, the fundamental structural components of silk fibroin, is related to H‐chains. H‐chains consist of 18 types of amino acids, that is, glycine (G, ∼45%), alanine (A, ∼30%), serine (S, ∼12%), tyrosine (Y, ∼5%), valine (V, ∼2%), and other 13 amino acids.^16^ Structurally, H‐chains comprise 12 hydrophobic repetitive domains interspersed with 11 hydrophilic non‐repetitive domains (Figure 1A). The hydrophobic domains containing glycine, alanine, and serine can self‐assemble into *ß*‐crystallites through intermolecular and/or intramolecular interactions.^15^ In detail, each repetitive domain comprises about five subdomains partitioned by tetrapeptides (GAAS), while each subdomain comprises diverse repetitive units of hexapeptides (GAGAGX, where X could represent alanine, serine, tyrosine, or valine) and terminates with a tetrapeptide (Figure 1B).^22^ The *n* hydrophilic domains consist primarily of charged or acidic amino acids assembled into the random coils and helical structures in natural silk fiber.^23^

### 3.2 Mechanical properties

Silk fibroin exhibits remarkable mechanical properties over other biomaterials due to its unique hierarchical structures, which are mainly determined by *ß*‐sheet crystallites. Native silk fibers have been reported to have a large breaking strain (10%–30%), strength (600 MPa), as well as toughness (70 MJ m^−3^), which are higher than many synthetic fibers.^24^, ^25^, ^26^ Benefiting from the outstanding strength and toughness, an increasing number of researchers have exploited silk fibroin as tissue engineering scaffold materials.^27^, ^28^ However, it should be noted that in most tissue engineering applications, silk fibroin‐derived scaffolds are usually processed from recombinant fibroin solution, and the resultant scaffolds show weak and brittle performances. Such defects could be ascribed to the chain hydrolysis of fibroin during degumming and dissolution as compared to raw silk fibers, thus leading to a lack of significant secondary and hierarchical structures.^24^, ^27^ Recently, the mechanical properties of recombinant fibroin have been significantly improved by various processing approaches, such as temperature adjustment, various agents' treatment, and even blended or cross‐linked with other polymer materials.^29^, ^30^, ^31^, ^32^

### 3.3 Biocompatibility

Native silkworm silk fibers have been utilized as sutures in various biomedical applications for decades.^33^, ^34^, ^35^, ^36^ Inevitably, the silk fibers show reactions ranging from delayed allergies to acute and chronic inflammatory processes in practical application.^37^ Sericin was previously regarded as the main participant in inducing the immune responses mounted against the fibers and ultimately leading to inflammations, such as T‐cell‐mediated anaphylactic reaction.^38^ Therefore, braided sutures are often degummed to reduce their inflammatory/immune response for clinical practice. After degumming, silk fibers can be processed into surgical nets, sutures, and clothing for various skin diseases.^33^ However, Kaplan et al. have revealed that fibroin and sericin fibers were immunoinert when co‐cultured with macrophages in vitro, while macrophages produced a stronger response when being co‐cultured with sericin‐coated fibroin.^39^ Such results suggested that the activation of macrophages was dependent on the physical binding of fibroin and sericin, in which the sericin‐coated fiber might provide improved adhesion for macrophages or the conformation of sericin changed during binding with silk fibroin that stimulates the macrophages. Thus, the use of silk as a biological material requires the separation of these two kinds of proteins to make sericin and silk fibroin independently possess great biocompatibility.

### 3.4 Biodegradability

Silk fibroin hydrogels have shown excellent in vitro and in vivo degradability.^40^ Enzymatic degradation promotes silk fibroin to fragment into smaller polypeptides and eventually become amino acids, so silk fibroin can also be considered bioabsorbable.^41^ Many proteolytic enzymes can trigger the degradation of silk fibroin hydrogels, such as protease XIV, proteinase K, α‐chymotrypsin, and papain, etc.^42^, ^43^ Among them, protease XIV is deemed as the most effective protease so far, which can degrade different silk fibroin‐derived products, including sponges, films, particles, as well as bulk materials, due to its many cleavage sites on silk fibroin chains allowing for more efficient degradation.^43^, ^44^ Therefore, protease XIV is widely used for the evaluation of in vitro degradation of silk fibroin.

Significantly, the degradation rate of silk fibroin hydrogels is highly dependent on the presence of *ß*‐sheet structure.^45^ It is noted that the native silk fibers show a lower degradation rate than regenerated silk fibroin fibers, which is ascribed to the higher content of *ß*‐sheet secondary structure of natural silk fibers than that of RSF structure. Lu et al. proposed that the hydrophilic bulk of silk fibroin was first degraded during the degradation process, and then the hydrophobic crystallites got rid of the surrounding and binding of the hydrophilic bulk to become free particles, followed by the movement toward the protease solution.^46^ Based on this degradation mechanism, the degradation process of silk fibroin is more controllable and flexible, making it a feasible solution to control the degradation rate of silk fibroin without sacrificing other excellent properties, which extensively expands the application of silk fibroin materials in biomedicine. In addition, silk fibroin's in vivo degradation behavior is related to the body's immune response, in which foreign body giant cells and macrophages perform a critical role in the degradation. Kaplan's team et al. revealed that 8 weeks after SF porous scaffolds were implanted in nude mice and Lewis rats, Lewis rats showed obvious signs of macrophage‐mediated degradation and lost structural integrity compared with nude mice.^47^

## 4 GELATION OF SILK FIBROIN HYDROGEL

**FIGURE 2:**
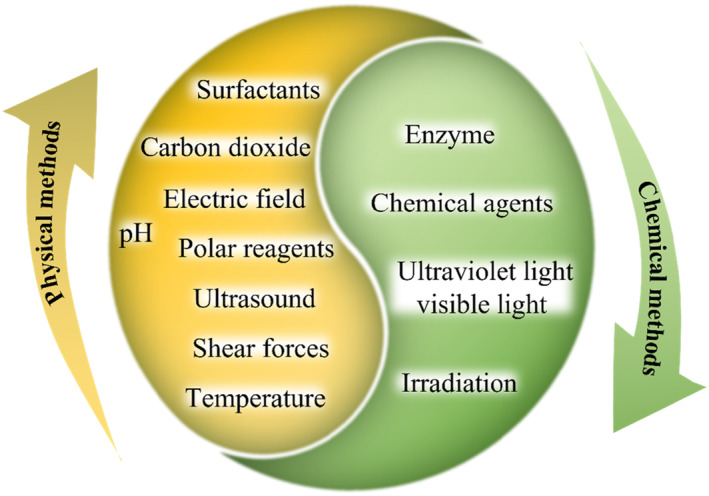
Overview of cross‐linking methods of silk fibroin hydrogel

Recently, increasing silk fibroin‐derived hydrogel products have emerged for various biomedical applications. According to the cross‐linking method, they could be classified into physically and chemically cross‐linked hydrogels (Figure 2 and Table 1).

**TABLE 1:** A summary of cross‐linking methods of silk fibroin hydrogels

Classification	Methods	Advantages	Disadvantages	Ref
Physical cross‐linking	Temperature	Mild and easy condition No toxic agents	Slow gelation	^48^, ^49^, ^51^, ^52^
	Shear forces	Simple operation No toxic agents Directional gel structures	Relatively high gelation concentration	^53^, ^54^, ^55^
	Ultrasound	Rapidity and controllability Environmental protection No toxic agents	High ultrasonic intensity	^56^, ^57^, ^58^, ^59^
	Polar reagents	Rapid and easy processing	Cytotoxicity	^60^, ^61^, ^62^, ^63^
	pH	Promoted gelation process	Acid condition Cytotoxicity	^48^
	Carbon dioxide	Directional gel structures No toxic agents	Slow gelation	^65^, ^66^
	Electric field	Free of residual acids or chemical cross‐linkers	Long processing time Stiff gel	^67^, ^68^
	Surfactants	Accelerated gelation	Release of surfactants	^70^, ^71^
Chemical cross‐linking	Enzymatic cross‐linking	Moderate reaction Biocompatibility Elastic gel structures	Time‐consuming	^72^, ^73^, ^74^, ^75^
	Chemical agents	Rapid gelation	Cytotoxicity (some agents)	^76^, ^77^, ^78^, ^79^, ^80^, ^81^, ^82^
	Photo cross‐linking	Efficient, rapid and, mild process	Cytotoxicity	^83^, ^84^, ^85^, ^86^
	Irradiation	Satisfactory rapidity	High energy consumption	^91^, ^92^

### 4.1 Physical cross‐linking

Silk fibroin solutions are able to transform into a hydrogel with 3D network structures via physical interactions.^48^ Such interactions occur under mild conditions dispensing with chemical cross‐linking agents, whose processing strategies include concentration adjustment, temperature raising, shear force applying, ultrasound trigger, pH change, organic solvent usage, surfactants exposure, salt adding, and electric field applying, etc.^49^, ^50^

#### 4.1.1 Temperature

For protein‐based materials, temperature is one of the key factors of gel formation. During the temperature‐triggered gelation process, on the one hand, increasing temperature promotes the frequency of effective molecular collisions in the system and then enhances the aggregation of silk fibroin molecules. Besides, the hydrophobic segment of silk fibroin increases with the temperature, thereby strengthening the hydrophobic interactions between molecules.^48^, ^50^, ^51^ Together, these events lead to the aggregation and self‐assembly of silk fibroin. In silk fibroin, the temperature‐trigged transition is irreversible because of the thermodynamically stable property of formed *ß*‐sheets. Generally, silk fibroin solutions could be stored at 4°C for around a week, while gelation occurs faster by placing solutions at room temperature.^49^, ^52^

#### 4.1.2 Shear forces

Significantly, silk fibroin exhibits great sensitivity to mechanical forces.^54^ Native fibroin solutions experience only subtle tensile and shear flow forces in the silk gland to prevent premature gelation of fibers prior to final spinning.^50^ Therefore, applying shear forces to the silk fibroin solutions in vitro can easily promote the *ß*‐sheet structure formation in silk fibroin hydrogels.^54^ Stirring and flowing are both able to induce the conformation transition of silk fibroin from disordered helical structures to ordered *ß*‐sheet structures. In addition, vortexing is a useful strategy to induce variation in shear gradients of fibroin structures, during which gel dynamics can be manipulated by changing the vortexing time and thus various time‐sensitive applications, such as cell encapsulation, are allowed.^55^ These properties lead to faster gelation of silk fibroin, and such a gelation approach is easily operated and requires no toxic reagents, making it an attractive green method.

#### 4.1.3 Ultrasound

Ultrasound can accelerate the intermolecular interactions of silk fibroin and induce its structural changes, thereby promoting rapid gelation.^56^, ^57^, ^58^ The changes caused by ultrasonication include local temperature rise, shear force extension, and gas–liquid interface balance change.^53^ Natalia Gorenkova et al. treated silk fibroin solution with ultrasound for 15–45 s to induce its gel formation.^59^ Benefiting from the advantages of rapidity, controllability, environmental protection, and nontoxic by‐products, the ultrasonic method is regarded as an effective physical method to prepare silk fibroin hydrogels. In the process of applying this method, the pregel solution concentration, ultrasonic output power, and action time are the principal elements for the resultant hydrogels.

#### 4.1.4 Polar reagents

To accelerate the physical gelation process, alcohols can usually be introduced to induce the *ß*‐sheet formation in silk fibroin hydrogels.^60^, ^61^, ^62^, ^63^, ^64^ The polarity of the solvent plays a significant role during the gelation process of silk fibroin using the mechanism that the polar reagents use to capture water molecules from the silk molecular chains, resulting in the formation of *ß*‐sheet nucleation sites.^53^ Notably, different alcohols show diverse induction effects on fibroin gelation. Alcohols containing longer fatty segments, such as n‐butanol, induce *ß*‐sheet formation in a milder manner.^60^ Although with rapid and easy processing, solvents' presence is one of the factors limiting cell applications (especially in situ cell encapsulation).

#### 4.1.5 Others

Adjusting pH value is also a familiar way to induce the gelation of silk fibroin. When the pH of the silk solution reaches its isoelectric point (pH 3.8–4.0), minimal charge repulsion occurs, and the silk fibroin nanoparticles remain on an unstable course, which is more easily aggregating to form a hydrogel.^48^ Particularly, adding carbon dioxide into the silk fibroin solution can also adjust the pH.^65^, ^66^ Besides, it is worth mentioning that applying an electric field can also act as an inducer for fibroin gelation.^67^ Under the applied electric field, the movement toward the anode of numerous protons in the solution results in a lower pH compared with the isoelectric point of the silk fibroin.^68^ As a result, the silk fibroin molecules show electronegativity so that they aggregate near the anode and finally form a hydrogel network. In addition, various surfactants represented by sodium dodecyl sulfate (SDS) can also be utilized to alter the silk gelation rate.^69^, ^70^ Adding surfactants can increase the hydrophobic interactions between silk molecules, as well as silk fibroin and surfactants, thus promoting the formation of *ß*‐sheet structures and accelerating the gelation process.^71^

### 4.2 Chemical cross‐linking

Although the physical cross‐linking method is simple to operate, requires no chemical reagents and has a mild reaction process, the physically cross‐linked hydrogels are often brittle and show poor mechanical properties. In contrast, the chemical cross‐linking method effectively overcomes these limitations, and the performances of derived hydrogels, such as mechanical strength, porosity, swelling degree, together with biodegradability, can be more flexibly tuned.

#### 4.2.1 Enzymatic cross‐linking

Enzymatic cross‐linked hydrogels have attracted considerable interest in biomedical applications owing to their moderate reaction conditions and remarkable biocompatibility. Commonly used enzymes include horseradish peroxidase (HRP), glutaminase, and tyrosinase.^32^, ^72^, ^73^, ^74^ Among them, HRP is the most popular enzymatic cross‐linking agent. Adding HRP/hydrogen peroxide (H_2_O_2_) to the silk fibroin solution can form a stable, highly elastic, and transparent hydrogel, which is due to the fact that HRP promotes the cross‐linking of tyrosine residues in fibroin molecular chains by forming free radicals in the presence of H_2_O_2_.^75^ The concentrations of HRP and H_2_O_2_ can control the mechanical properties of resultant silk fibroin hydrogels. Compared with physically cross‐linked and other chemical cross‐linking agent‐derived hydrogels, HRP/H_2_O_2_ catalyzed cross‐linking to prepare silk fibroin hydrogels shows a series of advantages. One of the significant features is the excellent biocompatibility and tunable biodegradability. In particular, the enzymatic cross‐linking can regulate the fragility of silk fibroin hydrogels while maintaining their elastic structures similar to ECM.

#### 4.2.2 Chemical agents

Chemical cross‐linking agents, such as genipin, glutaraldehyde, epoxide, and carbodiimide, can be covalently combined with the hydroxyl, amino, carboxyl, and other active groups in the silk fibroin molecule chains.^76^ Genipin is deemed as an excellent natural cross‐linking agent with lower toxicity than other conventional chemical cross‐linking agents.^77^ The cross‐linking reaction between genipin and silk fibroin is mainly related to lysine and arginine. Due to the low proportion of lysine and arginine in the silk fibroin, the genipin‐based cross‐linking process takes a long time.^78^ Glutaraldehyde can react with the ε‐amino and phenol group of lysine and tyrosine in fibroin, respectively, and its cross‐linking reaction with silk fibroin molecules has been confirmed.^79^ However, the cytotoxicity of glutaraldehyde limits its wide application. As for epoxide, it undergoes chemical cross‐linking reactions with amino acid residues such as lysine, tyrosine, histidine, and arginine on the side chain of silk fibroin.^80^ Additionally, 1‐(3‐Dimethylaminopropyl)‐3‐ethylcarbodiimide hydrochloride (EDC) can react with amino acids of silk fibroin with carboxyl side chains such as lysine, glutamic acid, and aspartic acid, so EDC is able to be utilized for the chemical cross‐linking reaction of silk fibroin.^81^ In particular, more stable amine‐reactive *N*‐Hydroxy succinimide (NHS) ester intermediates can be generated when adding NHS into the EDC reaction system, thereby improving the cross‐linking reaction efficiency.^82^

#### 4.2.3 Photo cross‐linking

Photo‐cross‐linking is a method that utilizes ultraviolet/visible light to trigger the polymerization process of materials. The first strategy to produce photo‐cross‐linked silk hydrogel is the formation of covalent bonds between reactive photoreactive groups (mainly tyrosine residues) of fibroin with the addition of reductants. Whittaker et al. first proposed a rapid and mild ruthenium (Ru)‐catalyzed photo‐cross‐linking strategy to generate silk fibroin hydrogels.^83^, ^84^ In fibroin solution, under the catalysis of tris(2,2‐bipyridyl) dichloro ruthenium (II) hexahydrate (Ru(II)(bpy)_3_^2+^), visible light induced the cross‐linking of silk fibroin via di‐tyrosine links to form hydrogels. Similar to Ru, riboflavin‐mediated photo‐cross‐linking is also achieved through the formation of di‐tyrosine bonds.^85^, ^86^ Besides, the second photo‐cross‐linking strategy is a chemical modification of silk fibroin chains. Methacryloyl allows efficient and rapid free radical polymerization; so methacryloyl modification has become a versatile processing method for the photo‐cross‐linked silk fibroin hydrogel. Recently, glycidyl methacrylate (GMA) has been mainly studied for the introduction of methacryloyl groups to silk fibroin.^87^, ^88^, ^89^, ^90^

#### 4.2.4 Irradiation

Irradiation technology has developed an advanced strategy to prepare hydrogels, because the polymer chains can easily undergo chemical cross‐linking and copolymerization under irradiation, and finally hydrogels are obtained. Such a process is controllable and simple, requires no additives such as initiators and cross‐linking agents, and shows satisfactory rapidity and economy. Gamma‐ray (γ‐ray) irradiation induces the generation of amounts of free radicals from polymer chains and water molecules, whose reorganization leads to intermolecular cross‐linking and forming of a hydrogel network.^91^ Recently, γ‐ray irradiation has been used to generate the silk fibroin hydrogel. From the research results of Kim et al., the secondary structure of the fibroin hydrogel formed by γ‐ray irradiation showed no change, which was still a random coil conformation, indicating that the hydrogel was formed by chemical cross‐linking reaction.^92^

It is worth mentioning that silk fibroin hydrogels can also be prepared by combining physical and chemical cross‐linking strategies.^53^ Kim et al. prepared silk fibroin hydrogels using photochemical cross‐linking and methanol‐induced physical cross‐linking.^93^ The pregel solution was exposed to visible light for 60 s to gel and then treated with 90% (v/v) methanol for 60 min. The resultant silk hydrogel showed high elasticity as well as outstanding structural stability. In another study, Su et al. reported a dual cross‐linked hydrogel by HRP/H_2_O_2_ pre‐cross‐linking and followed ethanol‐induced physical cross‐linking.^94^ Likewise, the resulting dual cross‐linked hydrogels were demonstrated to have enhanced mechanical properties and significantly increased stability. The aforementioned dual cross‐linked hydrogels consist of only a single silk fibroin network, wherein both chemical and physical cross‐links are present. In addition, double‐network hydrogels in which other material‐based hydrogel networks are introduced have also been proposed. Xiao and colleagues used ultrasonic and photopolymerization strategies to construct a double‐network hydrogel based on silk fibroin and methacrylated hyaluronic acid, showing high mechanical strength, high water content, and slow degradation rate.^95^

## 5 SILK FIBROIN HYDROGELS FOR TISSUE REGENERATION

Over the past decades, silk fibroin hydrogels have shown significant potential in multifarious tissue engineering applications, involving bone, cartilage, skin, nerve, vascular, ligament, tendon, liver, cornea, eardrum, dental, bladder, etc.^96^ In this chapter, we will focus on the applications of silk fibroin hydrogel on bone, cartilage, skin, and nerve regeneration applications.

### 5.1 Skin regeneration

**FIGURE 3:**
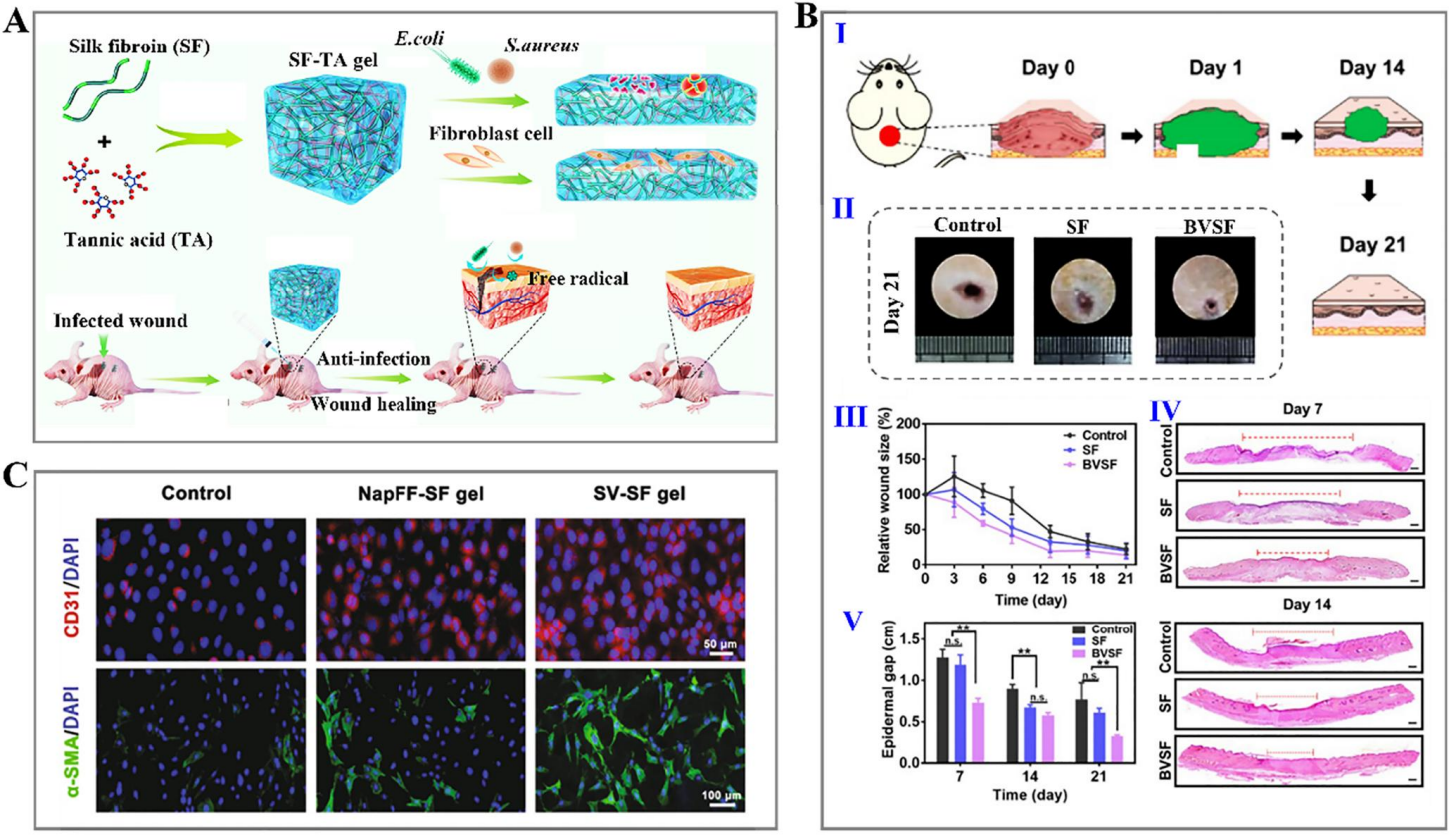
(A) Schematic illustration of silk fibroin and tannic acid (SF‐TA) hydrogel with high biocompatibility and bactericidal activity and its application as a wound dressing in a mouse model.^98^ (Reproduced with permission: Copyright 2019, American Chemical Society). (B) (I) Schematic illustration of the wound healing process with silk hydrogel treatment; (II) wound images of rats with different treatments after 21 days; (III) relative wound size change of different groups; (IV) hematoxylin‐eosin (H&E) staining of the wounds at 7 and 14 days; and (V) statistics of the epidermal gaps.^99^ (Reproduced under terms of the CC‐BY license: Copyright 2020, The Authors, published by Ivyspring International Publisher). (C) Immunofluorescence staining images of CD31 and α‐SMA by human umbilical vein endothelial cells (HUVECs) and bone marrow mesenchymal stem cells (BMSCs) that were planted on the different substrates.^100^ (Reproduced with permission: Copyright 2021, Elsevier).

As the largest organ of the human body, the skin is composed of the epidermis and dermis tightly combined and is the body's barrier against infectious microorganisms. Severe damage to the skin can result in loss of skin integrity, disability, and even death in severe cases. Silk fibroin hydrogels have been shown to support the attachment and migration of fibroblasts and keratinocytes and have been widely used for skin regeneration.^97^ Jing's group reported a hybrid hydrogel containing silk fibroin and tannic acid (SF‐TA) with remarkable antibacterial and antioxidant activity, which contributed to the accelerated wound healing of mice with full‐thickness skin defects (Figure 3A).^98^ Similarly, mixing silk fibroin with carboxymethyl chitosan (CMCS) can also yield hydrogel dressings with antibacterial properties. Silk fibroin/CMCS hydrogel sustained a proper microenvironment for wound healing and promoted re‐epithelialization and granulation tissue formation (Figure 3B).^99^ The development of biomaterials that can mimic the natural cellular environment in structure and function is also a primary objective of tissue engineering. In a recent study, a novel bioactive hydrogel comprising self‐assembling peptides and silk fibroin was designed for skin tissue engineering. Amphiphilic peptide could trigger rapid gelation of silk fibroin through cooperative self‐assembly and confer hydrogel ability to promote the adhesion, growth, and migration of endothelial cells. When implanted into a defected area, such a hydrogel could promote angiogenesis and epidermization, thereby achieving epidermal repair (Figure 3C).^100^ Furthermore, depending on the type of wound, functionalizing the hydrogel dressing with various signaling molecules or factors is another fascinating alternative. For example, the asiaticoside (AC) was loaded into silk fibroin nanofiber hydrogels to regulate inflammatory responses and angiogenesis, achieving scar‐free skin regeneration after implantation into full‐thickness wound defects.^101^ Furthermore, silver nanoparticles with antibacterial properties and glycyrrhizic acid (GA) with anti‐inflammatory properties were also introduced into the silk fibroin hydrogel matrix to generate a dressing for wound healing.^102^

### 5.2 Bone regeneration

**FIGURE 4:**
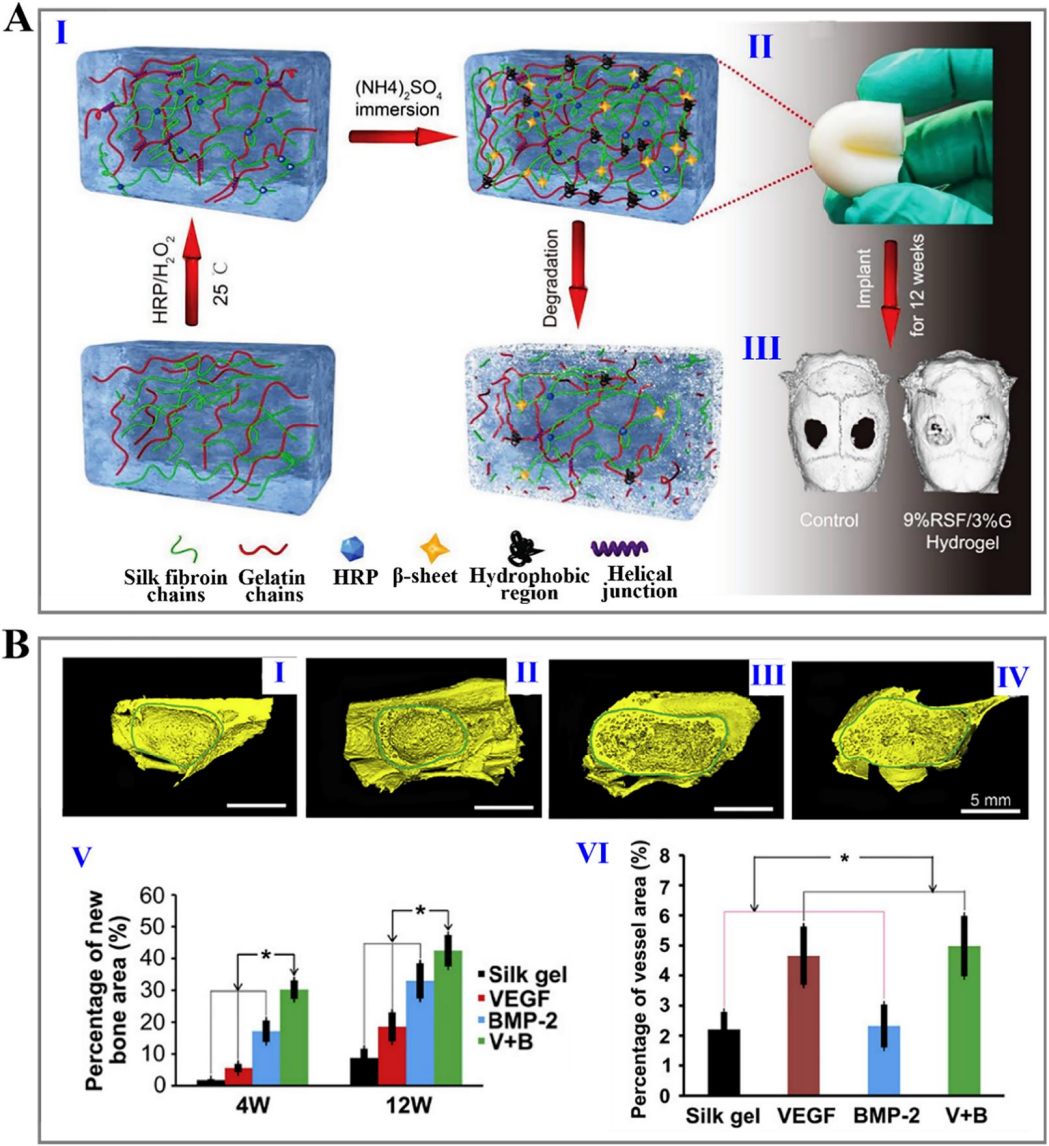
(A) (I) Schematic of the preparation of double‐cross‐linked silk fibroin hydrogels; (II) photographs of prepared hydrogels; and (III) representative micro‐CT images of the rat skull.^110^ (Reproduced with permission: Copyright 2019, John Wiley and Sons). (B) (I–IV) 3D reconstruction images of augmented sinus at 12 weeks postoperatively, which were treated by pure silk hydrogel (I), silk hydrogel loaded with VEGF (II), silk hydrogel loaded with BMP‐2 (III), and silk hydrogel loaded with = VEGF and BMP‐2 (V), respectively; (V, VI) Statistics of the bone formation (V) and new vessel area (VI).^111^ (Reproduced with permission: Copyright 2011, Elsevier). BMP‐2, bone morphogenetic protein‐2; VEGF, vascular endothelial growth factor.

Bone, as a special connective tissue, is mainly composed of type I collagen and hydroxyapatite, the nanocomposite structure of which contributes to the strength and hierarchical structure of bone tissue.^103^ Benefiting from their high toughness, robust mechanical strength, excellent biocompatibility, and slow biodegradability, silk fibroin hydrogel‐derived bone tissue engineering scaffolds have been developed as a very popular choice. Meinel et al. reported that porous silk fibroin‐based scaffolds precultured in a bioreactor for 5 weeks were implanted into defects of the mouse skull and successfully induced bone formation within 5 weeks.^104^ In addition, silk fibroin hydrogels can be combined with other biomaterials and therapeutic agents to enhance osteogenic properties.^105^, ^106^, ^107^, ^108^, ^109^ Jiang and coworkers presented silk fibroin/gelatin hydrogels with controllable strength and degradation rate, together with good biocompatibility and the capability to promote the proliferation and differentiation of BMSCs (Figure 4A).^110^ It was demonstrated that 12 weeks after being implanted into the skull defect, the silk fibroin/gelatin hybrid hydrogel could improve the bone regeneration quality and enhance the bone regeneration rate with less tissue response. In another study, Kim et al. used γ‐ray irradiation to fabricate hydroxyapatite nanoparticle (HAP NP)‐containing silk fibroin hydrogels for bone regeneration. They verified that the resultant silk fibroin/HAP NP composite hydrogel promoted osteogenic differentiation compared with pure silk fibroin hydrogels.^91^ It is important to note that thorough and fast vascularization is necessary to improve bone regeneration efficiency. Zhang and colleagues proposed an in situ formed silk hydrogel encapsulated with bone morphogenetic protein‐2 (BMP‐2) and vascular endothelial growth factor (VEGF165), aiming to promote new bone formation and angiogenesis (Figure 4B).^111^ These two elements showed synergistic effects on new bone formation, while an injectable silk hydrogel matrix was applied in a minimally invasive way to fill irregular bone defects.

### 5.3 Cartilage regeneration

**FIGURE 5:**
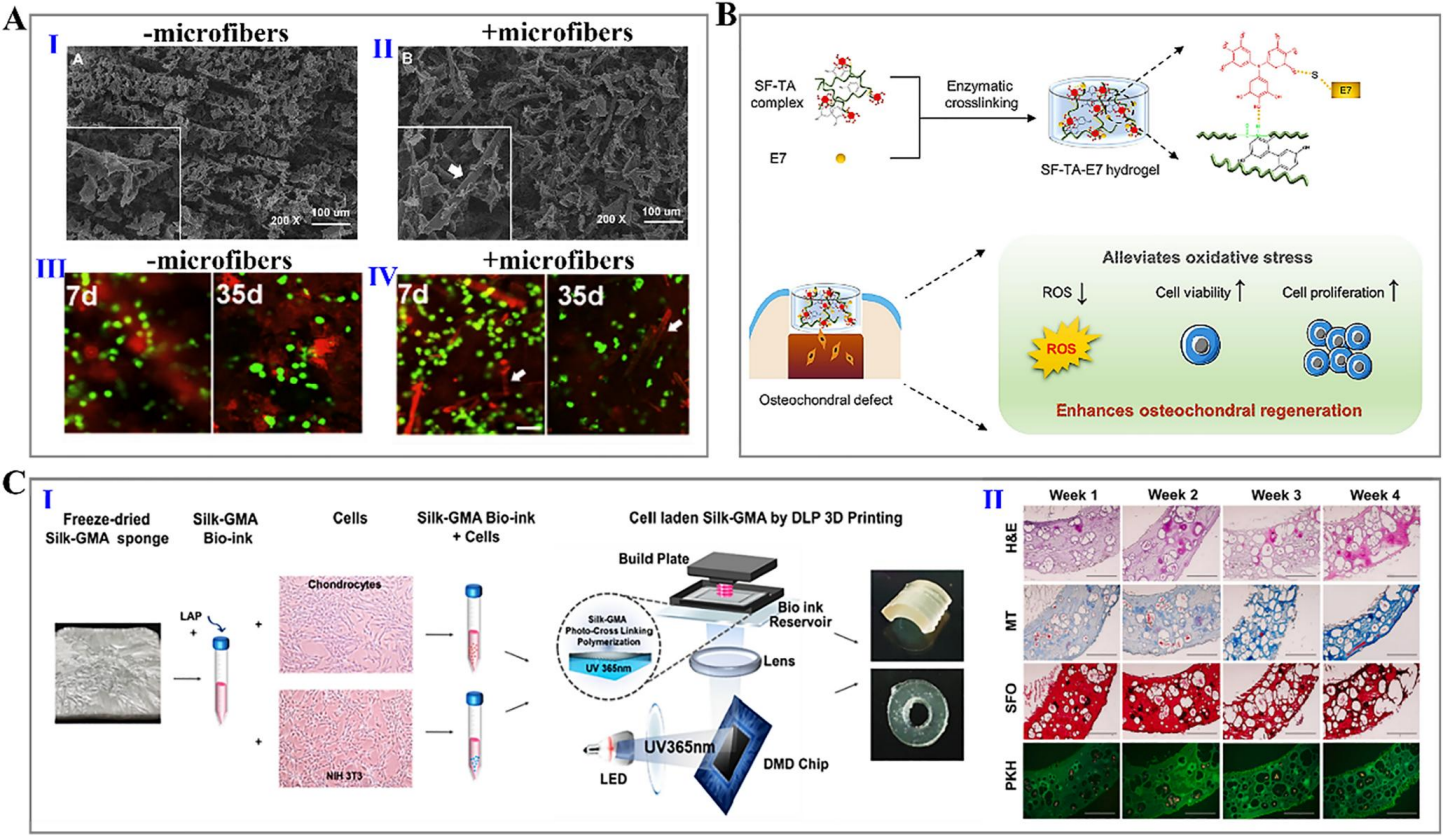
(A) (I, II) SEM images of prepared hydrogels without (I) and with (II) silk microfibers; (III, IV) live/dead images of cells cultured on the prepared hydrogels without (III) and with (IV) silk microfibers.^113^ (Reproduced with permission: Copyright 2015, Elsevier). (B) Schematic of the fabrication process and application of silk fibroin hydrogel.^114^ (Reproduced with permission: Copyright 2022, The Authors, published by Elsevier). (C) (I) Sketch of 3D printing process based on GMA‐modified silk fibroin hydrogels; (II) Histological detection of cartilage tissue treated with cell‐laden silk fibroin hydrogel.^28^ (Reproduced with permission: Copyright 2020, Elsevier). GMA, glycidyl methacrylate; SEM, Scanning electron microscope.

Cartilage is a supportive avascular connective tissue composed of chondrocytes and intercellular substances. Severe joint trauma, biomechanical imbalances, and degenerative changes can cause cartilage damage. Due to the limited self‐repairing ability of cartilage, these injuries often result in progressive damage and degeneration. Using the appropriate biomaterials as tissue engineering scaffolds is emerging as a potential alternative clinical strategy for cartilage repair. Among the numerous biomaterials used for osteochondral tissue engineering, silk fibroin hydrogels have attracted much attention owing to their outstanding physicochemical and biological performances. Researchers found that the silk fibroin hydrogel‐derived constructs showed functional properties matched with agarose‐based engineered cartilage.^112^ Subsequently, microfiber silk and ultrasound‐induced silk fibroin hydrogel were combined to develop a mechanically enhanced hydrogel for cartilage tissue engineering, which was observed with satisfactory chondrocyte response (Figure 5A).^113^ In particular, combining functional agents helps to improve the cartilage repair effect. Shen et al. used injectable silk fibroin hydrogels containing chondrocytes and exosomes to repair cartilage defects, effectively promoting cartilage regeneration in vivo.^107^ Zhang et al. proposed a multifunctional hydrogel composed of silk fibroin/TA for relieving oxidative stress and enhancing osteochondral regeneration, and bone marrow mesenchymal stem cells (BMSCs)‐specific affinity peptides were incorporated into hydrogels to recruit endogenous BMSCs (Figure 5B).^114^ As engineering technology develops, 3D printing technology has inspired a new wave of tissue engineering.^115^, ^116^, ^117^ Lee and workers constructed intact ear cartilage using silk fibroin and polyvinyl alcohol (PVA) composite hydrogels.^118^ The glycidyl methacrylate‐modified silk fibroin hydrogel prepared by Hong et al. based on 3D printing technology promoted the viability, proliferation, and differentiation into the cartilage of encapsulated cells for up to 4 weeks (Figure 5C).^28^ In the study by Li et al., 3D‐printed silk fibroin and gelatin hydrogel scaffolds were proposed, and enzymatic cross‐linking and methanol‐treated hybrid hydrogels combined with encapsulated cells could promote articular cartilage regeneration after 12 and 16 weeks of implantation.^119^

### 5.4 Nerve regeneration

**FIGURE 6:**
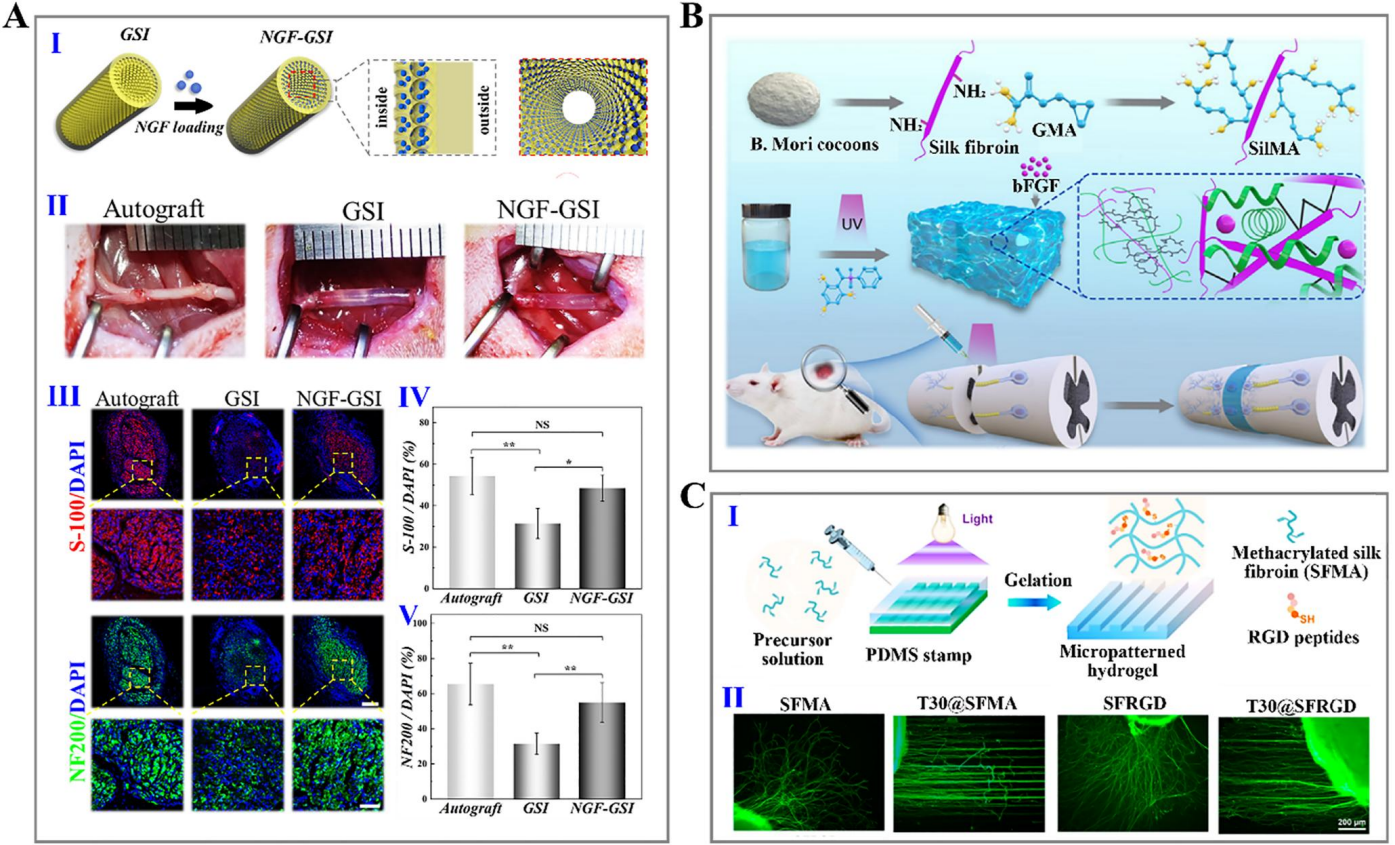
(A) (I) Schematic of fabricating silk nerve scaffolds with inverse opal structures, wherein GSI refers to the silk hydrogel conduit and NGF‐GSI refers to the NGF‐loaded silk hydrogel conduit; (II) representative images of transplanting surgery; and (III–V) immunofluorescence staining images (III) and statistics (IV, V) of S‐100 (Schwann cell‐specific marker) and NF200 (neurofilament marker) expressed by regenerated nerves.^120^ (Reproduced with permission: Copyright 2022, Elsevier). (B) Schematical illustration showing the modification of silk fibroin and the application of derived hydrogel on the repair of spinal cord injury.^121^ (Reproduced with permission: Copyright 2022, The Authors, published by Elsevier). (C) (I) Diagram of the fabrication process of micropatterned hydrogel; (II) immunofluorescence staining images of dorsal root ganglion (DRG) neurons cultured on the different substrates.^125^ (Reproduced with permission: Copyright 2021, American Chemical Society).

**FIGURE 7:**
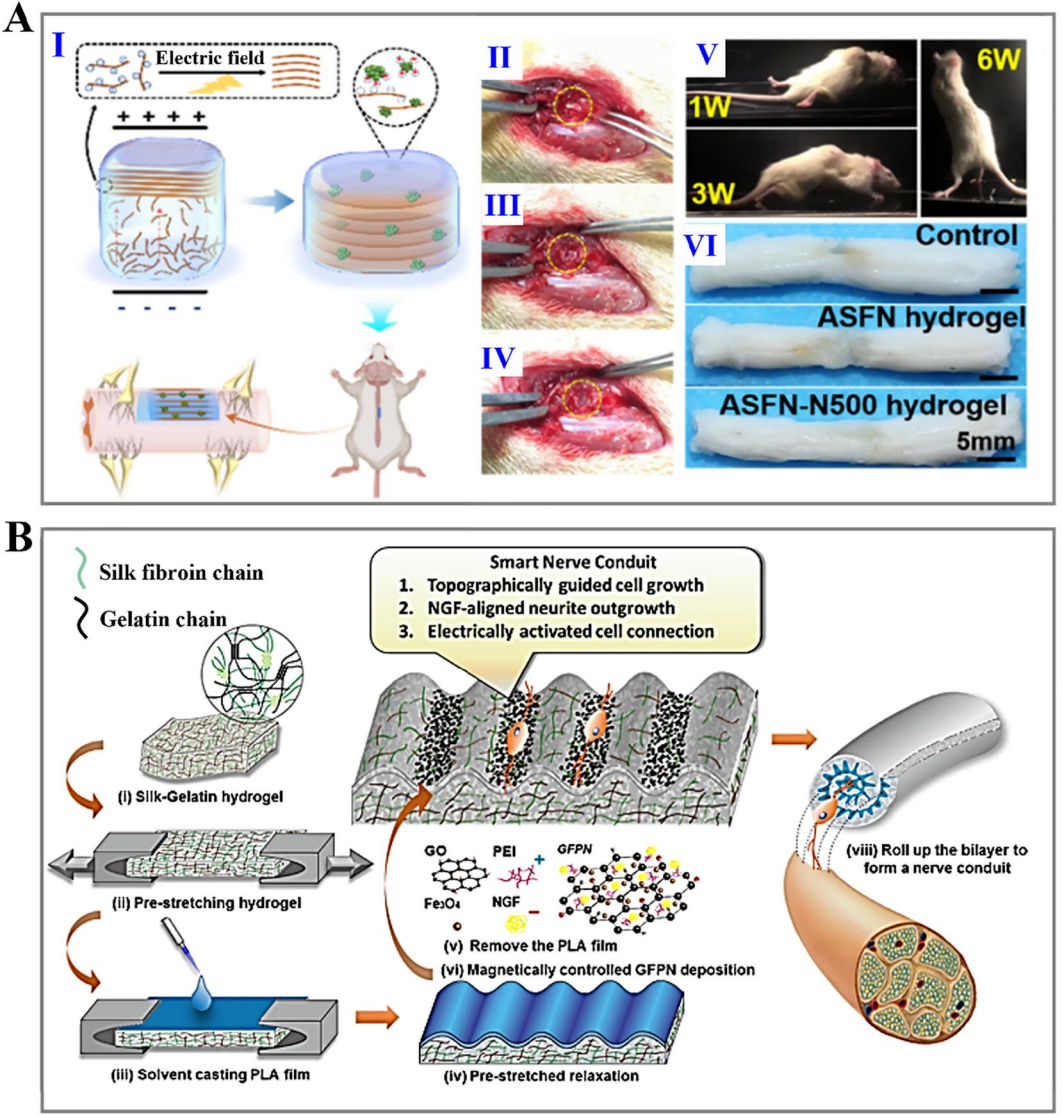
(A) (I) Schematical diagram of the aligned silk hydrogel applied for spinal cord injury treatment; (II–IV) representative images of surgery procedure; (V) photographs reflecting motor recovery of animals at different time points; and (VI) pictures of the spinal cord 6 weeks after surgery.^126^ (Reproduced with permission: Copyright 2022, American Chemical Society). (B) Sketch of the preparation process and application of smart nerve scaffold.^127^ (Reproduced with permission: Copyright 2020, American Chemical Society).

The human nervous system can fall into the central nervous system (CNS) and peripheral nervous system (PNS). PNS with minor injuries shows self‐repairing capability, while larger peripheral nerve injuries require autologous transplantation. In contrast, the repair of CNS injury is a complicated process due to endogenous factors inhibiting repair. Therefore, bioengineering approaches for PNS focus on replacements for nerve grafts, while solutions for CNS repair focus on creating a suitable environment to allow nerve regeneration. Silk fibroin hydrogels have been used to repair peripheral and central nerve injuries. Our recent work presented a novel silk fibroin hydrogel nerve conduit with great biocompatibility and suitable biodegradability, enabling targeted delivery of nerve growth factor (NGF) and efficient nerve repair (Figure 6A).^120^ Zhou et al. synthesized basic fibroblast growth factor (bFGF)‐loaded methacrylate‐silk fibroin hydrogels, which promoted neurite regeneration, inhibited glial cell proliferation, and improved neuronal mitochondrial function (Figure 6B).^121^ Furthermore, conductive hydrogels have emerged for nerve tissue regeneration due to their ability to mimic the biochemical and biophysical cues of the natural ECM, thereby enhancing the function of Schwann cells (SCs) and neurons.^122^ Zhao et al. fabricated the conductive biocomposite hydrogels containing silk and graphene oxide (GO) nanosheets. It was proved that the soft matrix contributed to SC survival and proliferation, while the increased conductivity favored the functional behavior of SCs.^123^ Importantly, topological microstructure design is also the focus of the nerve regeneration scaffolds. Gu et al. reported a pure silk fibroin hydrogel with a high‐strength and arrayed microgroove topography, showing a strong ability to promote directional outgrowth of axons and guide axonal sprouting.^124^ Tang et al. developed a silk‐based light‐triggered gel system with anisotropic topography and adhesion ligands, which achieved enhanced cell recruitment and myelination (Figure 6C).^125^ Besides, silk nanofiber hydrogels with aligned microstructures and NGF loaded provided dual physical and biological cues for promoting spinal cord regeneration (Figure 7A).^126^ More interestingly, Lin et al. fabricated a smart silk fibroin/gelatin hydrogel scaffold integrating topographic, biological, and synergistic effects of electrical stimulation to enhance neuronal activity. Such a multifunctional hydrogel device offered a predictable opportunity to develop novel nerve grafts (Figure 7B).^127^

## 6 SILK FIBROIN HYDROGELS FOR DRUG DELIVERY

**FIGURE 8:**
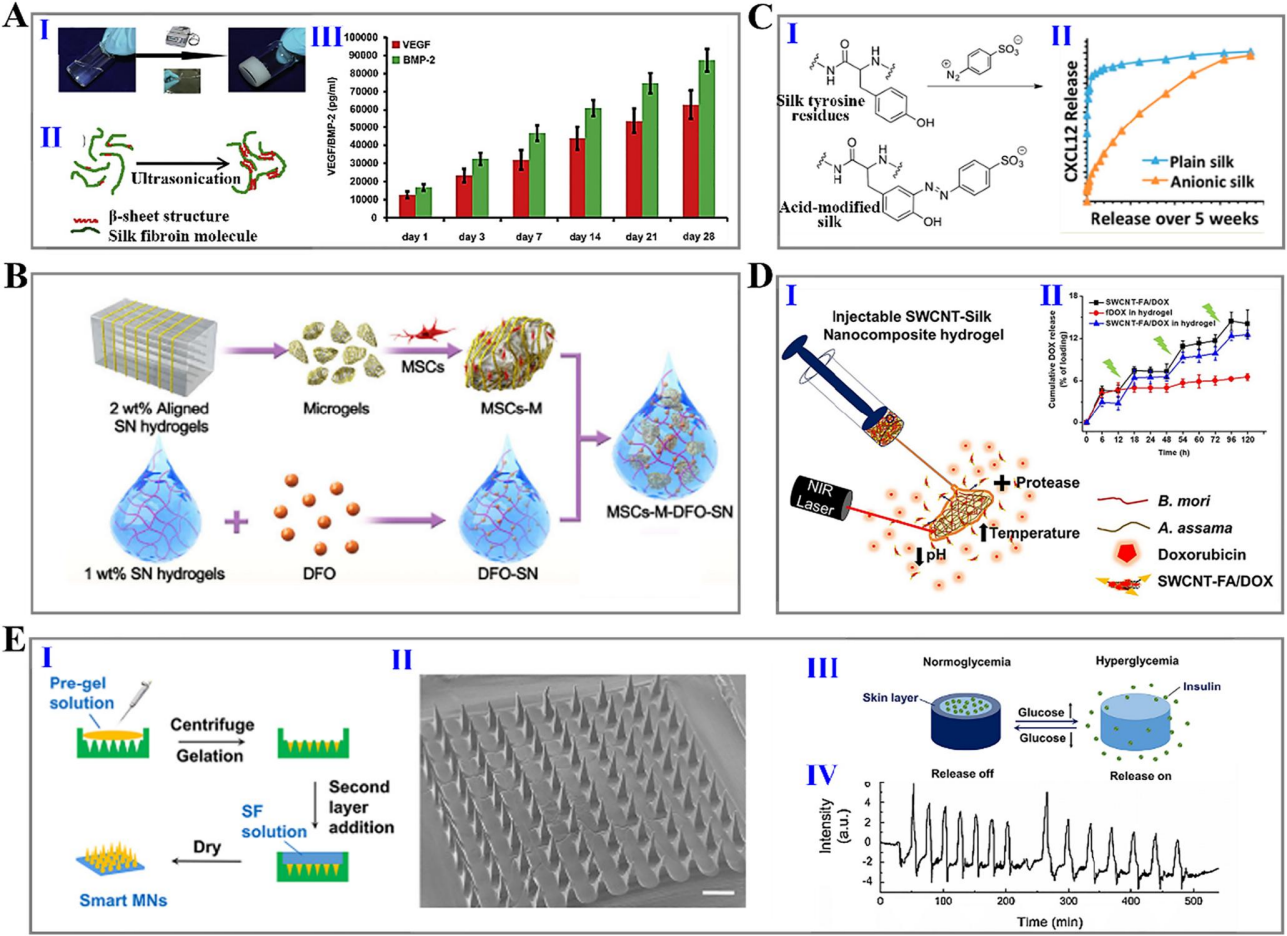
(A) (I) Photographs of silk hydrogel gelation process; (II) diagram of gelation mechanisms of silk hydrogel under ultrasonication; and (III) cumulative release of two drugs from hydrogel.^111^ (Reproduced with permission: Copyright 2011, Elsevier). (B) Diagram of generating silk hydrogel loaded with DFO and MSCs.^134^ (Reproduced with permission: Copyright 2022, John Wiley and Sons). (C) (I) Modification of silk with sulfonic acid; (II) cumulative rug release percentage from pure silk and modified silk hydrogels.^138^ (Reproduced with permission: Copyright 2015, American Chemical Society). (D) (I) Schematic of application of injectable carbon nanotube‐integrated silk hydrogel for on‐demand drug delivery; (II) cumulative drug release percentage from silk hydrogel under near‐infrared light trigger.^139^ (Reproduced with permission: Copyright 2019, American Chemical Society). (E) (I, II) Schematic representation of the fabrication process (I) and SEM image of smart MN (II); (III) schematic illustration of glucose‐responsive release of insulin; and (IV) fluorescence intensity change of FITC‐labeled insulin released from silk MN.^146^ (Reproduced with permission: Copyright 2019, American Chemical Society). DFO, deferoxamine; MSCs, mesenchymal stem cells; FITC, fluorescein isothiocyanate; MN, microneedle; SEM, Scanning electron microscope.

In the aspect of drug delivery, silk fibroin hydrogels are widely employed as drug delivery carriers due to their self‐assembly, processing flexibility, biocompatibility, biodegradability, as well as abilities to capture, retain, protect, and deliver therapeutic payloads.^19^, ^128^, ^129^, ^130^, ^131^ Zhang et al. presented an in situ formed silk fibroin hydrogel as a carrier to encapsulate bifactors. In vitro experiments presented that both of the loaded two factors showed no obviously explosive release, while in vivo tests proved an additive effect of VEGF165 and BMP‐2 released from the silk hydrogel on promoting angiogenesis and bone regeneration (Figure 8A).^111^ Sustained and slow delivery of the therapeutic payloads contributes to reducing the dosing frequency in clinical patients, thereby increasing their compliance.^132^ The processing parameters of silk fibroin hydrogels, such as the silk number, influence the silk vehicle performance. By reducing the content of silk fiber to 1% (wt), the drug release could be increased by a factor of 4.5 compared to the 6% (wt) hydrogel, which was attributed to the increased adsorption behavior of doxorubicin (DOX) on the silk hydrogel network structure with increasing *ß*‐sheet content in the hydrogel. This result indicated the controllable drug release behavior influenced by the silk content.^133^ In addition, the deferoxamine (DFO)‐loaded silk nanofiber hydrogels proposed by Kaplan's group achieved a sustained release of DFO over 40 days, which improved skin tissue repair by stimulating the formation of the vascular network (Figure 8B).^134^, ^135^ Besides, the modification of silk hydrogels shows an impact on their overall performance.^136^, ^137^ Atterberry et al. prepared hydrogels with ordinary silk fibroin and sulfonic acid‐modified silk fibroin.^138^ Compared with plain silk hydrogels, the modified hydrogels showed minimal burst and sustained release, which might be ascribed to the electrostatic interactions and hydrophobic effects (Figure 8C). Furthermore, many strategies have also been reported to improve the performance and drug delivery capacity of silk fibroin‐based hydrogels. For example, some other polymers or functional elements were involved in improving the physicochemical properties of silk fibroin hydrogels, or the drug‐loaded nanoparticles were introduced to further delay drug release (Figure 8D).^133^, ^139^, ^140^, ^141^, ^142^ Notably, microneedle (MN) technology provides a promising approach for drug delivery using noninvasive percutaneous treatment.^143^, ^144^, ^145^ Chen et al. reported a glucose‐responsive MN composed of silk fibroin and phenylboronic acid/acrylamide for smart insulin delivery (Figure 8E).^146^ The prepared MNs autonomously released insulin adapted to the glucose level changes.

Hitherto, Zhao's group has conducted numerous studies on the construction of drug delivery vehicles based on silk fibroin hydrogels. In 2019, we firstly reported silk fibroin hydrogel microspheres with inverse opal structures for drug delivery and postoperative treatment of tumors.^147^ On this basis, we combined the temperature‐responsive hydrogel (poly (*N*‐isopropylacrylamide), PNIPAM) with silk fibroin inverse opal microcarriers, achieving the controllable and sustained release of loaded drugs from carriers.^148^ Later, the introduction of functional elements, black phosphorus quantum dots, endowed silk fibroin inverse opal microspheres with photoresponsivity, while the secondary filling of gelatin with reversible phase transition capability enabled the sustained release of loaded drugs.^149^ The composite silk fibroin microcarriers have been demonstrated with the controlled drug delivery ability triggered by near‐infrared light, showing gratifying effect in wound repair. In addition to microspheres, we integrated inverse opal structures with tubular scaffolds to construct nerve conduits with localized delivery of growth factors, and in vitro and in vivo experiments confirmed their long‐term drug delivery ability.^120^ Overall, the silk fibroin hydrogels loaded with various therapeutic agents in different forms have shown positive outcomes on sustained, controllable, targeted, safe, and efficient delivery. We believe these works will provide new ideas for constructing silk fibroin‐based hydrogel delivery systems.

## 7 SILK FIBROIN HYDROGELS AS WEARABLE SENSORS

**FIGURE 9:**
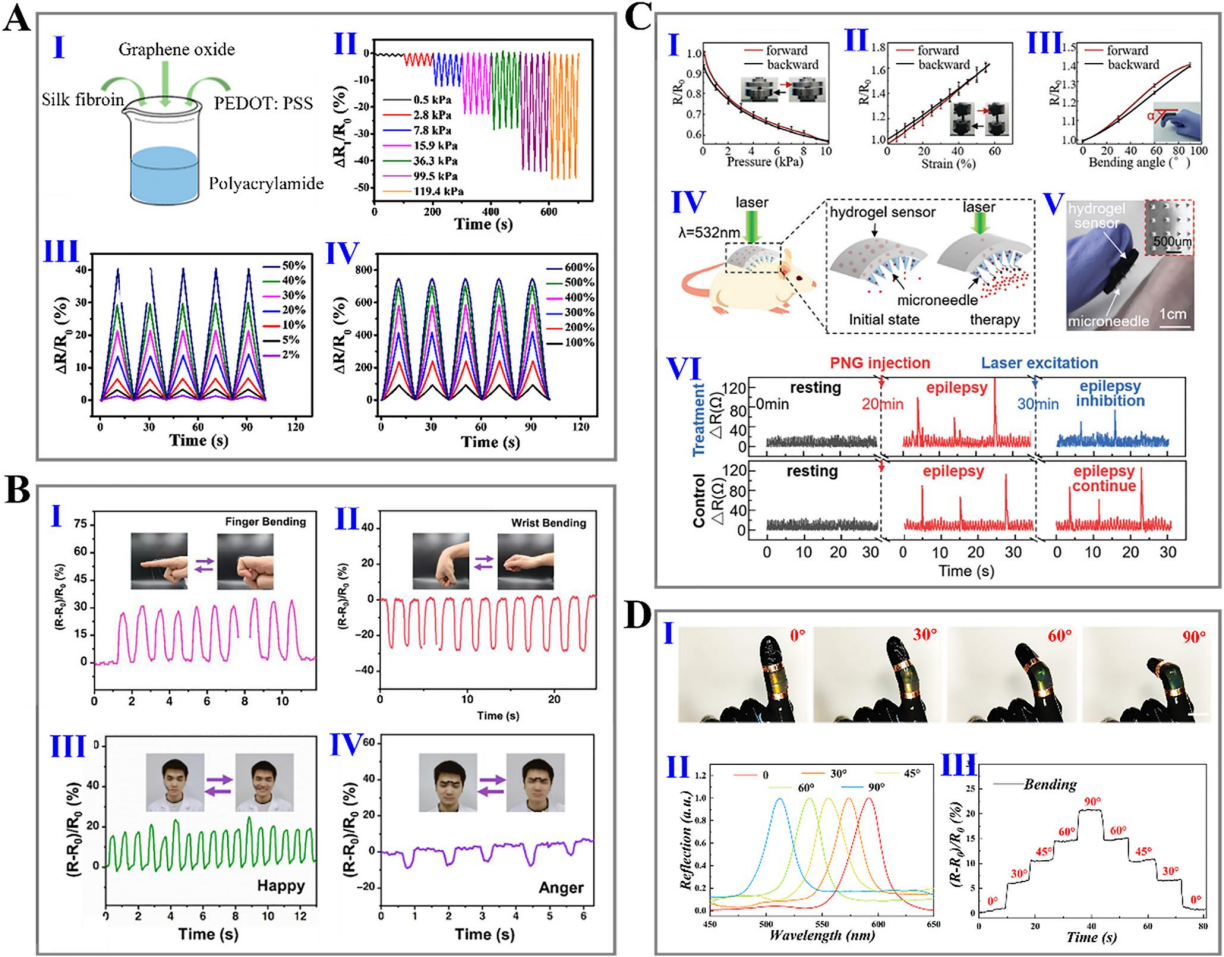
(A) (I) Schematic of composite silk fibroin hydrogel fabrication procedures; (II) electrical resistance changes under different pressures; and (III, IV) electrical resistance changes under different strains.^162^ (Reproduced with permission: Copyright 2020, American Chemical Society). (B) Electrical resistance changes in real‐time during different human motions: (I) finger bending; (II) wrist bending; (III) smiling; and (IV) frowning.^156^ (Reproduced with permission: Copyright 2022, Elsevier). (C) (I–III) Electrical resistance change under various treatments: pressure (I), tension strain (II), and bending (III); (IV) schematic diagram of silk‐based sensor integrated with silk microneedle as a smart drug delivery patch; (V) photograph of integrated silk patch; and (VI) detection and treatment of epilepsy according to the electrical resistance change.^163^ (Reproduced under terms of the CC‐BY license: Copyright 2020, Shanghai Institute of Microsystem and Information Technology, Chinese Academy of Sciences, published by John Wiley and Sons). (D) (I) Optical images of silk hydrogel under different bending angles; (II) reflection peak changes of silk hydrogel under different bending angles; and (III) relative resistance changes of silk hydrogel under different bending angles.^164^ (Reproduced under terms of the CC‐BY license: Copyright 2021, The Authors, published by John Wiley and Sons).

Wearable electronic devices have shown broad application potential in movement monitoring, personal medical and health care, as well as food hygiene and safety inspection, attracting people and increasing attention in recent years.^150^, ^151^ Wearable sensors are one of the key elements of flexible wearable electronic devices. With the rapid development of biomaterials and manufacturing processes, silk fibroin has become a promising substrate or sensing element for wearable sensor applications due to its remarkable biocompatibility, degradability, ease of processing, together with brilliant mechanical properties. Silk fibroin hydrogel‐based wearable sensors include mechanical (strain and pressure) sensors, humidity sensors, temperature sensors, electrophysiological sensors, and so on.^152^, ^153^, ^154^, ^155^, ^156^, ^157^, ^158^, ^159^, ^160^, ^161^ In a 2021 study, He et al. prepared a composite silk fibroin hydrogel strain/pressure sensor by mixing with polyacrylamide, graphene oxide, and poly(3,4ethylenedioxythiophene): poly(styrenesulfonate) (PEDOT: PSS) in proportions, which showed a wide sensing rage with 2%–600% strain and 0.5–119.4 kPa pressure (Figure 9A).^162^ Such a composite silk fibroin hydrogel sensor has been confirmed to monitor different human body signals. Through using silk fibroin, PVA, borax, and TA, Zheng et al. designed an ionically conductive hydrogel sensor, which can monitor changes of large strain movements, such as bending of finger joins and wrists, and could be utilized for sensitive and quick detection of micro‐deformation behaviors such as smiling, frowning, and breathing (Figure 9B).^156^ Uniquely, a type of mechanical sensor presented by Zhang et al. could not only detect variation in mechanical signals but also be integrated with drug‐loaded silk microneedles, showing satisfactory effects in continuous monitoring and treatment of epilepsy (Figure 9C).^163^ Remarkably, in our recent work, we combined silk fibroin hydrogels with inverse opal scaffolds to construct a composite hydrogel showing bright structural color and good electrical conductivity, stretchability, and flexibility (Figure 9D).^164^ The prepared hydrogel exhibited distinct structural color changes and resistance variation during the bending process, enabling it to be a visually wearable sensor with real‐time color sensing and electrical signal reporting capabilities.

**FIGURE 10:**
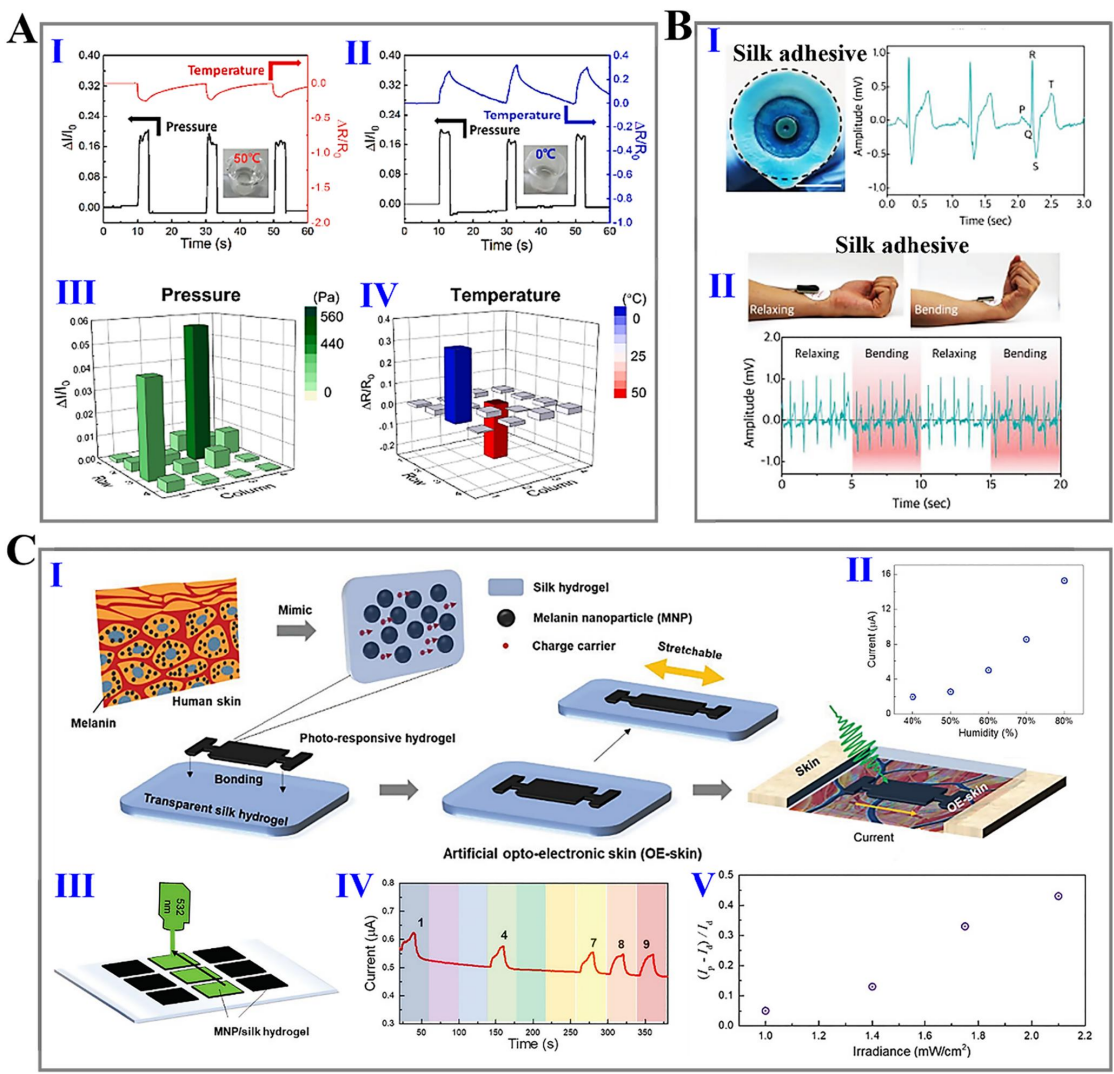
(A) (I, II) Pressure and temperature response of silk‐based sensor: (I) hot water, (II) cold water; and (III, IV) corresponding current response to pressure (III) and temperature (IV).^165^ (Reproduced with permission: Copyright 2021, Elsevier). (B) (I) Pictures of traditional electrodes (left) and ECG signals (right) measured with adhesive silk hydrogel; (II) photographs (top) and ECG signals (bottom) measured during bending and relaxing process with silk hydrogel.^167^ (Reproduced with permission: Copyright 2018, John Wiley and Sons). (C) (I) Schematic of working principle of prepared silk‐based OE skin; (II) real‐time current response of light connected to the silk‐based OE skin at different relative humilities; (III) schematic diagram of silk hydrogel array for laser‐lettering reading; (IV) real‐time current response to the letter writing process; and (V) photocurrent ratio of silk hydrogel under ultraviolet illumination with different irradiances.^168^ (Reproduced with permission: Copyright 2022, John Wiley and Sons). ECG, electrocardiogram; OE, optoelectronic.

In addition to the mechanical response, the real‐time feedback to the temperature variation of the body and surrounding environment is another vital ability of sensors.^165^, ^166^ Chen et al. embedded silver nanowires on the surface of silk fibroin hydrogels to prepare a pressure/temperature dual‐responsive sensor with excellent performances (Figure 10A).^165^ The temperature response test showed the high sensitivity and durability of the sensor from −30 to 50°C. In another report, Seo et al. proposed a calcium (Ca^2+^)‐modified silk fibroin hydrogel‐derived biosensor for electrophysiological signal monitoring (Figure 10B).^167^ Due to the incorporation of Ca^2+^, the mechanical interlocking of the interface between the silk fibroin hydrogel and the biological surface was enhanced, resulting in the robust adhesive property. Besides, its stretchability, electrical conductivity and reusability have also been confirmed. These properties make the resultant silk hydrogel useful as a multifunctional sensor. Particularly, the electrocardiogram (ECG) signals measured with silk fibroin adhesive were not distorted even while undergoing repeated bending and maintained comfortable contact with the skin, and thus ECG signals could be continuously and accurately recorded. In contrast, metal electrodes using commercial hydrogels (with lower peel strength) exhibited fluctuations in the measured signal during the bending process. In a recent study, Wahab et al. proposed a type of photoresponsive, self‐healing, and conductive optoelectronic (OE) skin using silk fibroin hydrogel and melanin nanoparticles (Figure 10C).^168^ Melanin nanoparticles with special photosensitive conductivity were incorporated into silk fibroin hydrogels and exhibited enhanced conductivity triggered by light irradiation. Such a novel OE skin showed humidity‐dependent conductivity, making it possible as a humidity sensor. In addition, illuminating the hydrogel region with a 532 nm green laser produced a higher current, which could be captured so the laser irradiation path could be derived to read out the encoded signal. Interestingly, OE skin could also be used as body‐integrated ultraviolet sensors to detect harmful ultraviolet rays, as the photocurrent ratio increased with the irradiated power density.

## 8 CHALLENGES AND PROSPECTS

Silk fibroin hydrogels possess excellent biocompatibility, tunable mechanical properties and degradation rates, thermal stability, and easy chemical modification, all of which make them extremely promising in biomedical fields. With the continuous deepening of researches on silk fibroin, the research process of new products based on silk fibroin hydrogel has been greatly accelerated. However, there are still some key issues that need to be explored in depth.

First, pure silk fibroin hydrogels have shown poor ability to attach and proliferate certain cells, and 3D cell cultivation has been limited. Therefore, a more comprehensive view of the relationship between silk fibroin hydrogels and various cells and tissues is required. Researchers need to consider combining silk fibroin with bioactive peptides or other ECM components, or modifying silk fibroin to enhance its applicability in biological applications. Various physical and chemical cross‐linking strategies have been outlined, but it is necessary to fully consider the influence of different gelation strategies on the viability of encapsulated cells and to optimize suitable processing tactics. In addition, the latest engineering manufacturing technologies, such as 3D printing, nanotechnology, genetic engineering, and even 4D printing technology, should be focused on exploring new silk fibroin hydrogel‐based biomaterials, aiming to meet specific needs in different situations. By regulating the secondary structure of silk fibroin, its mechanical performance and degradation rate can be optimized. For tissue engineering, it is especially necessary to design secondary silk structures so that the mechanical properties and degradation rate can match the new tissues. Most importantly, the current researches on silk fibroin hydrogels focus on cell and small animal models instead of large animal models, resulting in the slow clinical translation process. Therefore, more research studies are needed to develop products based on this excellent biomaterial, leading to clinical trials and FDA approval.

## AUTHOR CONTRIBUTIONS

Renjie Chai, Minli Li and Yong Zhang provided the idea; Hui Zhang, Dongyu Xu, Renjie Chai, Minli Li and Yong Zhang wrote and revised the manuscript.

## CONFLICT OF INTEREST

The authors declare no conflict of interest.

## References

[1] S. Correa , A. K. Grosskopf , H. Lopez Hernandez , D. Chan , A. C. Yu , L. M. Stapleton , E. A. Appel , Chem. Rev. 2021, 121, 11385. 33938724 10.1021/acs.chemrev.0c01177 PMC8461619

[2] Y. S. Zhang , A. Khademhosseini , Science 2017, 356, eaaf3627. 28473537 10.1126/science.aaf3627 PMC5841082

[3] H. Yuk , B. Lu , X. Zhao , Chem. Soc. Rev. 2019, 48, 1642. 30474663 10.1039/c8cs00595h

[4] C. Lin , A. T. Metters , Adv. Drug Deliv. Rev. 2006, 58, 1379. 17081649 10.1016/j.addr.2006.09.004

[5] D. Caccavo , S. Cascone , G. Lamberti , A. A. Barba , Chem. Soc. Rev. 2018, 47, 2357. 29504613 10.1039/c7cs00638a

[6] Y. Zhu , Q. Zhang , X. Shi , D. Han , Adv. Mater. 2019, 31, 1804950. 10.1002/adma.201804950 30815920

[7] D. Chimene , R. Kaunas , A. K. Gaharwar , Adv. Mater. 2020, 32, 1902026. 10.1002/adma.201902026 31599073

[8] A. C. Daly , L. Riley , T. Segura , J. A. Burdick , Nat. Rev. Mater. 2020, 5, 20. 34123409 10.1038/s41578-019-0148-6 PMC8191408

[9] R. Dimatteo , N. J. Darling , T. Segura , Adv. Drug Deliv. Rev. 2018, 127, 167. 29567395 10.1016/j.addr.2018.03.007 PMC6003852

[10] J. Wang , C. Shao , Y. Wang , L. Sun , Y. Zhao , Engineering 2020, 6, 1244.

[11] C. Shao , Y. Liu , J. Chi , F. Ye , Y. Zhao , Engineering 2021, 7, 1778.

[12] M. C. Catoira , L. Fusaro , D. Di Francesco , M. Ramella , F. Boccafoschi , J. Mater. Sci. Mater. Med. 2019, 30, 115. 31599365 10.1007/s10856-019-6318-7 PMC6787111

[13] J. Liu , X. Ge , L. Liu , W. Xu , R. Shao , Mater. Adv. 2022, 3, 2291.

[14] L. D. Koh , Y. Cheng , C. Teng , Y. W. Khin , X. J. Loh , S. Y. Tee , M. Low , E. Ye , H. Yu , Y. Zhang , M. Han , Prog. Polym. Sci. 2015, 46, 86.

[15] C. Wang , K. Xia , Y. Zhang , D. L. Kaplan , Acc. Chem. Res. 2019, 52, 2916. 31536330 10.1021/acs.accounts.9b00333

[16] W. Huang , S. Ling , C. Li , F. G. Omenetto , D. L. Kaplan , Chem. Soc. Rev. 2018, 47, 6486. 29938722 10.1039/c8cs00187a PMC6113080

[17] J. G. Hardy , T. R. Scheibel , Prog. Polym. Sci. 2010, 35, 1093.

[18] S. Bai , S. Liu , C. Zhang , W. Xu , Q. Lu , H. Han , D. L. Kaplan , H. Zhu , Acta Biomater. 2013, 9, 7806. 23628774 10.1016/j.actbio.2013.04.033

[19] S. Zhang , S. A. M. Shah , K. Basharat , S. A. Qamar , A. Raza , A. Mohamed , M. Bilal , H. M. N. Iqbal , J. Drug Deliv. Sci. Technol. 2022, 72, 103385.

[20] C. Zhou , F. Confalonieri , N. Medina , Y. Zivanovic , C. Esnault , T. Yang , M. Jacquet , J. Janin , M. Duguet , R. Perasso , Z. Li , Nucleic Acids Res. 2000, 28, 2413. 10871375 10.1093/nar/28.12.2413 PMC102737

[21] S. Inoue , K. Tanaka , F. Arisaka , S. Kimura , K. Ohtomo , S. Mizuno , J. Biol. Chem. 2000, 275, 40517. 10986287 10.1074/jbc.M006897200

[22] C. Zhou , F. Confalonieri , M. Jacquet , R. Perasso , Z. Li , J. Janin , PROTEINS: Struct. Funct. Genet. 2001, 44, 119. 11391774 10.1002/prot.1078

[23] H. Liu , Z. Sun , C. Guo , Adv. Fiber Mater. 2022, 4, 705.

[24] W. Sun , D. A. Gregory , M. A. Tomeh , X. Zhao , Int. J. Mol. Sci. 2021, 22, 1499. 33540895 10.3390/ijms22031499 PMC7867316

[25] J. M. Gosline , P. A. Guerette , C. S. Optlepp , K. N. Savage , J. Exp. Biol. 1999, 202, 3295. 10562512 10.1242/jeb.202.23.3295

[26] S. J. Blamires , P. T. Spicer , P. J. Flanagan , Front. Mater. 2020, 7, 29.

[27] B. Kundu , R. Rajkhowa , S. C. Kundu , X. Wang , Adv. Drug Deliv. Rev. 2013, 65, 457. 23137786 10.1016/j.addr.2012.09.043

[28] H. Hong , Y. B. Seo , D. Y. Kim , J. S. Lee , Y. J. Lee , H. Lee , O. Ajiteru , M. T. Sultan , O. J. Lee , S. H. Kim , C. H. Park , Biomaterials 2020, 232, 119679. 31865191 10.1016/j.biomaterials.2019.119679

[29] X. Tan , L. Liu , A. Mitryashkin , Y. Wang , J. C. H. Goh , ACS Biomater. Sci. Eng. 2022, 8, 3242. 35786841 10.1021/acsbiomaterials.2c00313

[30] Y. Guan , H. You , J. Cai , Q. Zhang , S. Yan , R. You , Carbohydr. Polym. 2020, 239, 116232. 32414432 10.1016/j.carbpol.2020.116232

[31] W. Shi , M. Sun , X. Hu , B. Ren , J. Cheng , C. Li , X. Duan , X. Fu , J. Zhang , H. Chen , Y. Ao , Adv. Mater. 2017, 29, 1701089. 10.1002/adma.201701089 28585319

[32] O. Hasturk , K. E. Jordan , J. Choi , D. L. Kaplan , Biomaterials 2020, 232, 119720. 31896515 10.1016/j.biomaterials.2019.119720 PMC7667870

[33] C. Holland , K. Numata , J. Rnjak‐Kovacina , F. P. Seib , Adv. Healthc. Mater. 2019, 8, 1800465. 10.1002/adhm.201800465 30238637

[34] N. Sivanesan , R. Venugopal , A. Subramanian , J. Ind. Tex. 2021, 51, 5202S.

[35] X. Wang , P. Liu , Q. Wu , Z. Zheng , M. Xie , G. Chen , J. Yu , X. Wang , G. Li , D. Kaplan , ACS Appl. Mater. Interf. 2022, 14, 11177. 10.1021/acsami.2c00106 35192338

[36] Y. Yang , X. Chen , F. Ding , P. Zhang , J. Liu , X. Gu , Biomaterials 2007, 28, 1643. 17188747 10.1016/j.biomaterials.2006.12.004

[37] B. Kundu , N. E. Kurland , S. Bano , C. Patra , F. B. Engel , V. K. Yadavalli , S. C. Kundu , Prog. Polym. Sci. 2014, 39, 251.

[38] Z. Fu , W. Li , J. Wei , K. Yao , Y. Wang , P. Yang , G. Li , Y. Yang , L. Zhang , ACS Biomater. Sci. Eng. 2022, 8, 1494. 35230824 10.1021/acsbiomaterials.1c01426

[39] B. Panilaitis , G. H. Altman , J. Chen , H. J. Jin , V. Karageorgiou , D. L. Kaplan , Biomaterials 2003, 24, 3079. 12895580 10.1016/s0142-9612(03)00158-3

[40] B. Liu , Y. Song , L. Jin , Z. Wang , D. Pu , S. Lin , C. Zhou , H. You , Y. Ma , J. Li , L. Yang , K. L. Sung , Y. Zhang , Colloids Surf. B Biointerfaces 2015, 131, 122. 25982316 10.1016/j.colsurfb.2015.04.040

[41] Y. Cao , B. Wang , Int. J. Mol. Sci. 2009, 10, 1514. 19468322 10.3390/ijms10041514 PMC2680630

[42] M. Li , M. Ogiso , N. Minoura , Biomaterials 2003, 24, 357. 12419638 10.1016/s0142-9612(02)00326-5

[43] C. Guo , C. Li , D. L. Kaplan , Biomacromolecules 2020, 21, 1678. 32040910 10.1021/acs.biomac.0c00090 PMC7645160

[44] L. Chambre , R. N. Parker , B. J. Allardyce , F. Valente , R. Rajkhowa , R. J. Dilley , X. Wang , D. L. Kaplan , ACS Appl. Bio Mater. 2020, 3, 2466. 10.1021/acsabm.0c00186 35025296

[45] S. Lu , X. Tang , Q. Lu , J. Huang , X. You , F. Zhang , Mater. Today Commun. 2021, 27, 102369.

[46] Q. Lu , B. Zhang , M. Li , B. Zuo , D. L. Kaplan , Y. Huang , H. Zhu , Biomacromolecules 2011, 12, 1080. 21361368 10.1021/bm101422j PMC3404841

[47] Y. Wang , D. D. Rudym , A. Walsh , L. Abrahamsen , H. J. Kim , H. S. Kim , C. Kirker‐Head , D. L. Kaplan , Biomaterials 2008, 29, 3415. 18502501 10.1016/j.biomaterials.2008.05.002 PMC3206261

[48] H. Zheng , B. Zuo , J. Mater. Chem. B 2021, 9, 1238. 33406183 10.1039/d0tb02099k

[49] M. Farokhi , M. Aleemardani , A. Solouk , H. Mirzadeh , A. H. Teuschl , H. Redl , Biomed. Mater. 2021, 16, 022004. 33594992 10.1088/1748-605X/abb615

[50] U. J. Kim , J. Park , C. Li , H. J. Jin , R. Valluzzi , D. L. Kaplan , Biomacromolecules 2004, 5, 786. 15132662 10.1021/bm0345460

[51] C. Yang , S. Li , X. Huang , X. Chen , H. Shan , X. Chen , L. Tao , M. Zhang , Oxid. Med. Cell. Longev. 2022, 2022, 2076680. 35547640 10.1155/2022/2076680 PMC9085322

[52] Z. Yin , F. Wu , T. Xing , V. K. Yadavalli , S. C. Kundu , S. Lu , RSC Adv. 2017, 7, 24085.

[53] Y. Zhao , Z. S. Zhu , J. Guan , S. J. Wu , Acta Biomater. 2021, 125, 57. 33601067 10.1016/j.actbio.2021.02.018

[54] Y. Zhang , Y. Zuo , S. Wen , Y. Hu , Y. Min , Biomacromolecules 2018, 19, 1223. 29481061 10.1021/acs.biomac.8b00070

[55] N. Kojic , E. M. Pritchard , H. Tao , M. A. Brenckle , J. P. Mondia , B. Panilaitis , F. Omenetto , D. L. Kaplan , Adv. Funct. Mater. 2012, 22, 3793. 24015118 10.1002/adfm.201200382 PMC3760432

[56] B. Cai , H. Gu , F. Wang , K. Printon , Z. Gu , X. Hu , Ultrason. Sonochem. 2021, 79, 105800. 34673337 10.1016/j.ultsonch.2021.105800 PMC8560629

[57] A. T. Nguyen , Q. Huang , Z. Yang , N. Lin , G. Xu , X. Liu , Small 2015, 11, 1039. 25510895 10.1002/smll.201402985

[58] S. K. Samal , D. L. Kaplan , E. Chiellini , Macromol. Mater. Eng. 2013, 298, 1201.

[59] N. Gorenkova , I. Osama , F. P. Seib , H. V. O. Carswell , ACS Biomater. Sci. Eng. 2018, 5, 859. 33405845 10.1021/acsbiomaterials.8b01024

[60] K. Kaewprasit , T. Kobayashi , S. Damrongsakkul , J. Appl. Polym. Sci. 2019, 137, 48731.

[61] M. A. de Moraes , C. R. Albrecht Mahl , M. Ferreira Silva , M. M. Beppu , J. Appl. Polym. Sci. 2015, 132, 41802.

[62] H. Chen , M. Lin , Y. Lai , B. Chen , J. Taiwan Inst. Chem. Eng. 2021, 129, 256.

[63] S. S. Silva , N. M. Oliveira , M. B. Oliveira , D. P. S. da Costa , D. Naskar , J. F. Mano , S. C. Kundu , R. L. Reis , Acta Biomater. 2016, 32, 178. 26766632 10.1016/j.actbio.2016.01.003

[64] M. Garcia‐Fuentes , E. Giger , L. Meinel , H. P. Merkle , Biomaterials 2008, 29, 633. 17996295 10.1016/j.biomaterials.2007.10.024

[65] M. L. Floren , S. Spilimbergo , A. Motta , C. Migliaresi , Biomacromolecules 2012, 13, 2060. 22657735 10.1021/bm300450a

[66] W. H. Elliott , W. Bonani , D. Maniglio , A. Motta , W. Tan , C. Migliaresi , ACS Appl. Mater. Interfaces 2015, 7, 12099. 25978549 10.1021/acsami.5b02308 PMC4872633

[67] Q. Lu , S. Bai , Z. Ding , H. Guo , Z. Shao , H. Zhu , D. L. Kaplan , Adv. Mater. Interfaces 2016, 3, 1500687.

[68] N. Kojic , M. J. Panzer , G. G. Leisk , W. K. Raja , M. Kojic , D. L. Kaplan , Soft Matter 2012, 8, 6897. 10.1039/C2SM25783A PMC3399521 22822409

[69] S. Hirlekar , D. Ray , V. K. Aswal , A. Prabhune , A. Nisal , S. Ravindranathan , Langmuir 2019, 35, 14870. 31625756 10.1021/acs.langmuir.9b02402

[70] J. H. Park , M. H. Kim , L. Jeong , D. Cho , O. H. Kwon , W. H. Park , J. Sol‐Gel Sci. Technol. 2014, 71, 364.

[71] Z. Li , Z. Zheng , Y. Yang , G. Fang , J. Yao , Z. Shao , X. Chen , ACS Sustain. Chem. Eng. 2016, 4, 1500.

[72] T. V. Chirila , S. Suzuki , C. Papolla , Biotechnol. Appl. Biochem. 2017, 64, 771. 28220960 10.1002/bab.1552

[73] N. R. Raia , B. P. Partlow , M. McGill , E. P. Kimmerling , C. E. Ghezzi , D. L. Kaplan , Biomaterials 2017, 131, 58. 28376366 10.1016/j.biomaterials.2017.03.046 PMC5479139

[74] B. Zhou , P. Wang , L. Cui , Y. Yu , C. Deng , Q. Wang , X. Fan , Appl. Biochem. Biotechnol. 2017, 182, 1548. 28138929 10.1007/s12010-017-2417-4

[75] M. Goczkowski , M. Gobin , M. Hindie , R. Agniel , V. Larreta‐Garde , Mater. Sci. Eng. C 2019, 104, 109931. 10.1016/j.msec.2019.109931 31499978

[76] N. Johari , L. Moroni , A. Samadikuchaksaraei , Eur. Polym. J. 2020, 134, 109842.

[77] R. You , Y. Xu , G. Liu , Y. Liu , X. Li , M. Li , Polymer. Degrad. Stabil. 2014, 109, 226.

[78] A. Vasconcelos , A. C. Gomes , A. Cavaco‐Paulo , Acta Biomater. 2012, 8, 3049. 22546517 10.1016/j.actbio.2012.04.035

[79] S. Kanokpanont , S. Damrongsakkul , J. Ratanavaraporn , P. Aramwit , Int. J. Pharm. 2012, 436, 141. 22771972 10.1016/j.ijpharm.2012.06.046

[80] M. Tsukada , M. Nagura , H. Ishkawa , H. Shiozaki , J. Appl. Polym. Sci. 1991, 43, 643.

[81] H. Niu , J. Xiao , X. Lou , L. Guo , Y. Zhang , R. Yang , H. Yang , S. Wang , F. Niu , Front. Bioeng. Biotechnol. 2022, 10, 800830. 35350178 10.3389/fbioe.2022.800830 PMC8957943

[82] Y. Wang , X. Wang , J. Shi , R. Zhu , J. Zhang , Z. Zhang , D. Ma , Y. Hou , F. Lin , J. Yang , M. Mizuno , Sci. Rep. 2016, 6, 39477. 27996001 10.1038/srep39477 PMC5172375

[83] J. L. Whittaker , N. R. Choudhury , N. K. Dutta , A. Zannettino , J. Mater. Chem. B 2014, 2, 6259. 32262143 10.1039/c4tb00698d

[84] J. L. Whittaker , N. K. Dutta , A. Zannettino , N. R. Choudhury , J. Mater. Chem. B 2016, 4, 5519. 32263350 10.1039/c6tb01055e

[85] M. B. Applegate , B. P. Partlow , J. Coburn , B. Marelli , C. Pirie , R. Pineda , D. L. Kaplan , F. G. Omenetto , Adv. Mater. 2016, 28, 2417. 26821561 10.1002/adma.201504527

[86] D. Kuang , F. Jiang , F. Wu , K. Kaur , S. Ghosh , S. C. Kundu , S. Lu , Int. J. Biol. Macromol. 2019, 134, 838. 31103592 10.1016/j.ijbiomac.2019.05.068

[87] I. A. Barroso , K. Man , V. M. Villapun , S. C. Cox , A. K. Ghag , ACS Biomater. Sci. Eng. 2021, 7, 4779. 34586800 10.1021/acsbiomaterials.1c00791

[88] S. H. Kim , Y. K. Yeon , J. M. Lee , J. R. Chao , Y. J. Lee , Y. B. Seo , M. T. Sultan , O. J. Lee , J. S. Lee , S. I. Yoon , I. S. Hong , G. Khang , S. J. Lee , J. J. Yoo , C. H. Park , Nat. Commun. 2018, 9, 1620. 29693652 10.1038/s41467-018-03759-y PMC5915392

[89] S. H. Kim , H. Hong , O. Ajiteru , M. T. Sultan , Y. J. Lee , J. S. Lee , O. J. Lee , H. Lee , H. S. Park , K. Y. Choi , J. S. Lee , H. W. Ju , I. S. Hong , C. H. Park , Nat. Protoc. 2021, 16, 5484. 34716451 10.1038/s41596-021-00622-1

[90] S. H. Kim , Y. J. Lee , J. R. Chao , D. Y. Kim , M. T. Sultan , H. J. Lee , J. M. Lee , J. S. Lee , O. J. Lee , H. Hong , H. Lee , O. Ajiteru , Y. J. Suh , H. S. Choi , Y. J. Cho , C. H. Park , NPG Asia Mater. 2020, 12, 46.

[91] M. H. Kim , B. S. Kim , J. Lee , D. Cho , O. H. Kwon , W. H. Park , Biomater. Res. 2017, 21, 12. 28652926 10.1186/s40824-017-0098-2 PMC5483289

[92] M. H. Kim , W. H. Park , Int. J. Nanomed. 2016, 11, 2967. 10.2147/IJN.S106467 PMC4922766 27382283

[93] C. S. Kim , Y. J. Yang , S. Y. Bahn , H. J. Cha , NPG Asia Mater. 2017, 9, e391.

[94] D. Su , M. Yao , J. Liu , Y. Zhong , X. Chen , Z. Shao , ACS Appl. Mater. Interfaces 2017, 9, 17489. 28470062 10.1021/acsami.7b04623

[95] W. Xiao , X. Qu , J. Li , L. Chen , Y. Tan , K. Li , B. Li , X. Liao , Eur. Polymer J. 2019, 118, 382.

[96] D. G. Harkin , K. A. George , P. W. Madden , I. R. Schwab , D. W. Hutmacher , T. V. Chirila , Biomaterials 2011, 32, 2445. 21251709 10.1016/j.biomaterials.2010.12.041

[97] D. Chouhan , T. U. Lohe , P. K. Samudrala , B. B. Mandal , Adv. Healthc. Mater. 2018, 7, 1801092. 10.1002/adhm.201801092 30379407

[98] J. Jing , S. Liang , Y. Yan , X. Tian , X. Li , ACS Biomater. Sci. Eng. 2019, 5, 4601. 33448833 10.1021/acsbiomaterials.9b00604

[99] Q. Yao , Q. Lan , X. Jiang , C. Du , Y. Zhai , X. Shen , H. Xu , J. Xiao , L. Kou , Y. Zhao , Theranostics 2020, 10, 11719. 33052243 10.7150/thno.47682 PMC7545989

[100] L. Wang , Z. Chen , Y. Yan , C. He , X. Li , Chem. Eng. J. 2021, 418, 129308.

[101] L. Liu , Z. Ding , Y. Yang , Z. Zhang , Q. Lu , D. L. Kaplan , Biomater. Sci. 2021, 9, 5227. 34190240 10.1039/d1bm00904d PMC8319114

[102] F. Zhang , C. Yin , X. Qi , C. Guo , X. Wu , Macromol. Biosci. 2022, 22, 2100407. 10.1002/mabi.202100407 34939312

[103] J. Melke , S. Midha , S. Ghosh , K. Ito , S. Hofmann , Acta Biomater. 2016, 31, 1. 26360593 10.1016/j.actbio.2015.09.005

[104] L. Meinel , R. Fajardo , S. Hofmann , R. Langer , J. Chen , B. Snyder , G. Vunjak‐Novakovic , D. Kaplan , Bone 2005, 37, 688. 16140599 10.1016/j.bone.2005.06.010

[105] B. Wang , S. Yuan , W. Xin , Y. Chen , Q. Fu , L. Li , Y. Jiao , Int. J. Biol. Macromol. 2021, 192, 407. 34597700 10.1016/j.ijbiomac.2021.09.036

[106] A. Zheng , L. Cao , Y. Liu , J. Wu , D. Zeng , L. Hu , X. Zhang , X. Jiang , Carbohydr. Polymer. 2018, 199, 244. 10.1016/j.carbpol.2018.06.093 30143127

[107] K. Shen , A. Duan , J. Cheng , T. Yuan , J. Zhou , H. Song , Z. Chen , B. Wan , J. Liu , X. Zhang , Y. Zhang , R. Xie , F. Liu , W. Fan , Q. Zuo , Acta Biomater. 2022, 143, 173. 35202856 10.1016/j.actbio.2022.02.026

[108] Y. Cheng , G. Cheng , C. Xie , C. Yin , X. Dong , Z. Li , X. Zhou , Q. Wang , H. Deng , Z. Li , Adv. Healthc. Mater. 2021, 10, 2001646. 10.1002/adhm.202001646 33694330

[109] Y. Jin , B. Kundu , Y. Cai , S. C. Kundu , J. Yao , Colloids Surf. B Biointerfaces 2015, 134, 339. 26209967 10.1016/j.colsurfb.2015.07.015

[110] L. Jiang , D. Su , S. Ding , Q. Zhang , Z. Li , F. Chen , W. Ding , S. Zhang , J. Dong , Adv. Funct. Mater. 2019, 29, 1901314.

[111] W. Zhang , X. Wang , S. Wang , J. Zhao , L. Xu , C. Zhu , D. Zeng , J. Chen , Z. Zhang , D. L. Kaplan , X. Jiang , Biomaterials 2011, 32, 9415. 21889205 10.1016/j.biomaterials.2011.08.047 PMC3384686

[112] P. H. Chao , S. Yodmuang , X. Wang , L. Sun , D. L. Kaplan , G. Vunjak‐Novakovic , J. Biomed. Mater. Res. B Appl. Biomater. 2010, 95, 84. 20725950 10.1002/jbm.b.31686 PMC3079331

[113] S. Yodmuang , S. L. McNamara , A. B. Nover , B. B. Mandal , M. Agarwal , T. A. Kelly , P. H. Chao , C. Hung , D. L. Kaplan , G. Vunjak‐Novakovic , Acta Biomater. 2015, 11, 27. 25281788 10.1016/j.actbio.2014.09.032 PMC4256092

[114] W. Zhang , Y. Zhang , X. Li , Z. Cao , Q. Mo , R. Sheng , C. Ling , J. Chi , Q. Yao , J. Chen , H. Wang , Mater. Today Bio 2022, 14, 100251. 10.1016/j.mtbio.2022.100251 PMC9034395 35469254

[115] X. Wang , C. Yang , Y. Yu , Y. Zhao , Research 2022, 2022, 9794745. 35387266 10.34133/2022/9794745 PMC8961369

[116] C. Li , W. Cui , Eng. Regen. 2021, 2, 195.

[117] S. Chawla , S. Midha , A. Sharma , S. Ghosh , Adv. Healthc. Mater. 2018, 7, 1701204. 10.1002/adhm.201701204 29359861

[118] J. M. Lee , M. T. Sultan , S. H. Kim , V. Kumar , Y. K. Yeon , O. J. Lee , C. H. Park , Int. J. Mol. Sci. 2017, 18, 1707. 28777314 10.3390/ijms18081707 PMC5578097

[119] Q. Li , S. Xu , Q. Feng , Q. Dai , L. Yao , Y. Zhang , H. Gao , H. Dong , D. Chen , X. Cao , Bioact. Mater. 2021, 6, 3396. 33842736 10.1016/j.bioactmat.2021.03.013 PMC8010633

[120] H. Zhang , H. Zhang , H. Wang , Y. Zhao , R. Chai , Appl. Mater. Today 2022, 27, 101431.

[121] L. Zhou , Z. Wang , D. Chen , J. Lin , W. Li , S. Guo , R. Wu , X. Zhao , T. Lin , G. Chen , W. Liu , Mater. Des. 2022, 217, 110670.

[122] Y. Cai , Q. Huang , P. Wang , K. Ye , Z. Zhao , H. Chen , Z. Liu , H. Liu , H. Wong , M. Tamtaji , K. Zhang , F. Xu , G. Jin , L. Zeng , J. Xie , Y. Du , Z. Hu , D. Sun , J. Qin , X. Lu , Z. Luo , Adv. Healthc. Mater. 2022, 11, 2200755. 10.1002/adhm.202200755 35670309

[123] Y. Zhao , J. Liu , Y. Gao , Z. Xu , C. Dai , G. Li , C. Sun , Y. Yang , K. Zhang , J. Mater. Chem. B 2022, 10, 1582. 35156678 10.1039/d1tb02361f

[124] X. Gu , X. Chen , X. Tang , Z. Zhou , T. Huang , Y. Yang , J. Ling , Nanotechnol. Rev. 2021, 10, 10.

[125] X. Tang , X. Gu , T. Huang , X. Chen , Z. Zhou , Y. Yang , J. Ling , ACS Macro Lett. 2021, 10, 1501. 35549152 10.1021/acsmacrolett.1c00533

[126] X. Gao , W. Cheng , X. Zhang , Z. Zhou , Z. Ding , X. Zhou , Q. Lu , D. L. Kaplan , ACS Appl. Mater. Interfaces 2022, 14, 3701. 35006667 10.1021/acsami.1c19229

[127] C. Lin , J. Chang , M. Yung , W. Huang , S. Chen , ACS Biomater. Sci. Eng. 2020, 6, 1144. 33464846 10.1021/acsbiomaterials.9b01449

[128] K. Numata , D. L. Kaplan , Adv. Drug Deliver. Rev. 2010, 62, 1497. 10.1016/j.addr.2010.03.009 PMC2901564 20298729

[129] H. Wu , S. Liu , L. Xiao , X. Dong , Q. Lu , D. L. Kaplan , ACS Appl. Mater. Interfaces 2016, 8, 17118. 27315327 10.1021/acsami.6b04424

[130] J. Wu , J. K. Sahoo , Y. Li , Q. Xu , D. L. Kaplan , J. Control. Release 2022, 345, 176. 35157939 10.1016/j.jconrel.2022.02.011 PMC9133086

[131] A. Florczak , T. Deptuch , K. Kucharczyk , H. Dams‐Kozlowska , Cancers 2021, 13, 5389. 34771557 10.3390/cancers13215389 PMC8582423

[132] M. L. Lovett , X. Wang , T. Yucel , L. York , M. Keirstead , L. Haggerty , D. L. Kaplan , Eur. J. Pharm. Biopharm. 2015, 95, 271. 25592326 10.1016/j.ejpb.2014.12.029

[133] F. P. Seib , E. M. Pritchard , D. L. Kaplan , Adv. Funct. Mater. 2013, 23, 58. 23646041 10.1002/adfm.201201238 PMC3639434

[134] L. Wu , K. Tian , Z. Ding , T. Zhao , X. Zhang , W. Cheng , S. Gao , Q. Lu , D. L. Kaplan , Adv. Ther. 2022, 5, 2100231.

[135] Z. Ding , M. Zhou , Z. Zhou , W. Zhang , X. Jiang , X. Lu , B. Zuo , Q. Lu , D. L. Kaplan , ACS Biomater. Sci. Eng. 2019, 5, 4077. 33448809 10.1021/acsbiomaterials.9b00621

[136] J. Kundu , L. A. Poole‐Warren , P. Martens , S. C. Kundu , Acta Biomater. 2012, 8, 1720. 22285428 10.1016/j.actbio.2012.01.004

[137] R. Yu , Y. Yang , J. He , M. Li , B. Guo , Chem. Eng. J. 2021, 417, 128278.

[138] P. N. Atterberry , T. J. Roark , S. Y. Severt , M. L. Schiller , J. M. Antos , A. R. Murphy , Biomacromolecules 2015, 16, 1582. 25894928 10.1021/acs.biomac.5b00144

[139] A. Gangrade , B. B. Mandal , ACS Biomater. Sci. Eng. 2019, 5, 2365. 33405786 10.1021/acsbiomaterials.9b00416

[140] B. B. Mandal , S. Kapoor , S. C. Kundu , Biomaterials 2009, 30, 2826. 19203791 10.1016/j.biomaterials.2009.01.040

[141] P. Wu , Q. Liu , Q. Wang , H. Qian , L. Yu , B. Liu , R. Li , Int. J. Nanomed. 2018, 13, 5405. 10.2147/IJN.S166104 PMC6149978 30271137

[142] M. Akrami‐Hasan‐Kohal , M. Eskandari , A. Solouk , Colloids Surf. B Biointerfaces 2021, 205, 111892. 34107443 10.1016/j.colsurfb.2021.111892

[143] X. Zhang , Y. Wang , J. Chi , Y. Zhao , Research 2020, 2020, 7462915. 33623910 10.34133/2020/7462915 PMC7877383

[144] X. Zhang , G. Chen , Y. Yu , L. Sun , Y. Zhao , Research 2020, 2020, 3672120. 32490376 10.34133/2020/3672120 PMC7231261

[145] X. Zhang , G. Chen , L. Cai , L. Fan , Y. Zhao , Research 2022, 2022, 9797482. 35958112 10.34133/2022/9797482 PMC9343079

[146] S. Chen , H. Matsumoto , Y. Moro‐Oka , M. Tanaka , Y. Miyahara , T. Suganami , A. Matsumoto , ACS Biomater. Sci. Eng. 2019, 5, 5781. 33405670 10.1021/acsbiomaterials.9b00532

[147] H. Zhang , Y. Liu , G. Chen , H. Wang , C. Chen , M. Li , P. Lu , Y. Zhao , Sci. Bull. 2020, 65, 380. 10.1016/j.scib.2019.10.023 36659229

[148] H. Zhang , Y. Liu , C. Chen , W. Cui , C. Zhang , F. Ye , Y. Zhao , Appl. Mater. Today 2020, 19, 100540.

[149] H. Zhang , Z. Zhang , H. Zhang , C. Chen , D. Zhang , Y. Zhao , ACS Appl. Mater. Interfaces 2021, 13, 18413. 33856190 10.1021/acsami.0c19884

[150] J. Guo , Y. Yu , D. Zhang , H. Zhang , Y. Zhao , Research 2021, 2021, 7065907. 33763650 10.34133/2021/7065907 PMC7953990

[151] Y. Yu , J. Guo , L. Sun , X. Zhang , Y. Zhao , Research 2019, 2019, 6906275. 31549079 10.34133/2019/6906275 PMC6750041

[152] X. Sun , S. He , M. Yao , X. Wu , H. Zhang , F. Yao , J. Li , J. Mater. Chem. C 2021, 9, 1880.

[153] F. Chen , S. Lu , L. Zhu , Z. Tang , Q. Wang , G. Qin , J. Yang , G. Sun , Q. Zhang , Q. Chen , J. Mater. Chem. B 2019, 7, 1708. 32254912 10.1039/c8tb02445f

[154] X. Liu , J. Liu , J. Wang , T. Wang , Y. Jiang , J. Hu , Z. Liu , X. Chen , J. Yu , ACS Appl. Mater. Interfaces 2020, 12, 5601. 31927972 10.1021/acsami.9b21197

[155] S. Li , G. Liu , H. Wen , G. Liu , H. Wang , M. Ye , Y. Yang , W. Guo , Y. Liu , Adv. Funct. Mater. 2022, 32, 2111747.

[156] H. Zheng , M. Chen , Y. Sun , B. Zuo , Chem. Eng. J. 2022, 446, 136931.

[157] B. Zhou , Y. Li , Y. Chen , C. Gao , J. Li , Z. Bai , J. Guo , Chem. Eng. J. 2022, 446, 137405.

[158] T. Chu , H. Wang , Y. Qiu , H. Luo , B. He , B. Wu , B. Gao , Analyst 2021, 146, 1552. 33475623 10.1039/d0an02292f

[159] J. Wang , N. Zhang , Y. Tan , F. Fu , G. Liu , Y. Fang , X. Zhang , M. Liu , Y. Cheng , J. Yu , ACS Appl. Mater. Interfaces 2022, 14, 21945. 35507426 10.1021/acsami.2c02534

[160] L. Zhao , J. Zhao , F. Zhang , Z. Xu , F. Chen , Y. Shi , C. Hou , Y. Huang , C. Lin , R. Yu , W. Guo , Adv. Healthc. Mater. 2021, 10, 2002083. 10.1002/adhm.202002083 33763942

[161] H. Zheng , N. Lin , Y. He , B. Zuo , ACS Appl. Mater. Interfaces 2021, 13, 40013. 34375080 10.1021/acsami.1c08395

[162] F. He , X. You , H. Gong , Y. Yang , T. Bai , W. Wang , W. Guo , X. Liu , M. Ye , ACS Appl. Mater. Interfaces 2020, 12, 6442. 31935061 10.1021/acsami.9b19721

[163] S. Zhang , Z. Zhou , J. Zhong , Z. Shi , Y. Mao , T. H. Tao , Adv. Sci. 2020, 7, 1903802. 10.1002/advs.201903802 PMC7341100 32670755

[164] H. Zhang , J. Guo , Y. Wang , L. Sun , Y. Zhao , Adv. Sci. 2021, 8, 2102156. 10.1002/advs.202102156 PMC8529447 34436831

[165] Q. Chen , H. Tang , J. Liu , R. Wang , J. Sun , J. Yao , Z. Shao , X. Chen , Chem. Eng. J. 2021, 422, 130091.

[166] C. Wang , M. Zhu , H. Yu , S. Y. H. Abdalkarim , Z. Ouyang , J. Zhu , J. Yao , ACS Appl. Mater. Interfaces 2021, 13, 33371. 34236852 10.1021/acsami.1c08568

[167] J. W. Seo , H. Kim , K. Kim , S. Q. Choi , H. J. Lee , Adv. Funct. Mater. 2018, 28, 1800802.

[168] A. Wahab , N. Gogurla , J. Y. Park , S. Kim , Adv. Mater. Technol. 2022, 7, 2101271.

